# TET2 directs mammary luminal cell differentiation and endocrine response

**DOI:** 10.1038/s41467-020-18129-w

**Published:** 2020-09-15

**Authors:** Mi Ran Kim, Meng-Ju Wu, Yingsheng Zhang, Jer-Yen Yang, Chun Ju Chang

**Affiliations:** 1 School of Sciences, Arts & Education, Ivy Tech Community College, Lafayette, IN USA; 2 Center for Cancer Research, Massachusetts General Hospital, Boston, MA USA; 3 Departments of Medicine, Harvard Medical School, Boston, MA USA; 4 Department of Medicine and Biological Sciences, Cedars-Sinai Medical Center, Los Angeles, CA USA; 5 Cedars-Sinai Samuel Oschin Comprehensive Cancer Institute, Los Angeles, CA USA; 6 Graduate Institute of Biomedical Sciences, College of Medicine, China Medical University, Taichung, 40402 Taiwan; 7 Research Center for Cancer Biology, China Medical University, Taichung, 40402 Taiwan; 8 Department of Medicine, Roswell Park Comprehensive Cancer Center, Buffalo, NY 19263 USA

**Keywords:** Breast cancer, Mammary stem cells

## Abstract

Epigenetic regulation plays an important role in governing stem cell fate and tumorigenesis. Lost expression of a key DNA demethylation enzyme TET2 is associated with human cancers and has been linked to stem cell traits in vitro; however, whether and how TET2 regulates mammary stem cell fate and mammary tumorigenesis in vivo remains to be determined. Here, using our recently established mammary specific Tet2 deletion mouse model, the data reveals that TET2 plays a pivotal role in mammary gland development and luminal lineage commitment. We show that TET2 and FOXP1 form a chromatin complex that mediates demethylation of *ESR1, GATA3,* and *FOXA1*, three key genes that are known to coordinately orchestrate mammary luminal lineage specification and endocrine response, and also are often silenced by DNA methylation in aggressive breast cancers. Furthermore, Tet2 deletion-PyMT breast cancer mouse model exhibits enhanced mammary tumor development with deficient ERα expression that confers tamoxifen resistance in vivo. As a result, this study elucidates a role for TET2 in governing luminal cell differentiation and endocrine response that underlies breast cancer resistance to anti-estrogen treatments.

## Abstract (web summary)

TET2 loss is associated with human cancers but its role in the mammary gland development and tumorigenesis is unclear. Here, the authors show that TET2–FOXP1 complex mediates demethylation of genes involved in luminal lineage commitment and endocrine response, underlying a role of TET2 loss in endocrine resistant breast cancer.

## Introduction

Stem cells are critical for tissue homeostasis and can serve as cells of origin of human cancers^1^. The mouse mammary gland has been widely recognized as an excellent model to study adult stem cells due to its dynamic plasticity and well-defined cell lineage hierarchy^2^. Specifically, mammary stem cells (MaSC), a specific sub-population of mammary epithelial cells that give rise to progenitor cells and their mature progenies, are able to generate an entire mammary gland that comprises all lineages, including the inner layer of ductal and alveolar luminal cells, and the outer layer of basal/myoepithelial cells^3^. Notably, dysregulation of cell fate decision and lineage commitment has been linked to breast tumorigenesis^4^; therefore, understanding how lineage differentiation is regulated will not only provide mechanistic insights into the maintenance of mammary epithelial homeostasis, but will have important implications for breast cancer pathogenesis and development of effective anti-cancer treatments.

In response to intrinsic queues or extrinsic stimuli, epigenetic regulation plays a crucial role in programming cell fate decisions through maneuvering global gene expression changes^5^. Accumulated evidence has revealed that a major chromatin modifier, ten-eleven translocation (TET), which mediates conversion of 5-methylcytosine (5mc) into 5-hydroxymethylcytosine (5hmC) to activate DNA demethylation, plays a critical role in governing embryonic and adult stem cell homeostasis^6,7^. Particularly, among the TET family proteins (TET1–3), TET2 is the most predominantly expressed in the mammary tissue (The Human Protein Atlas), and its expression is often silenced post-transcriptionally in human cancers^6,8,9^. Study has shown that repressed TET2 expression is linked to promoted epithelial-mesenchymal-transition (EMT) phenotype and expansion of a breast cancer stem cell-like population with skewed asymmetric cell division in vitro^10^; however, the in vivo role that TET2 plays in regulation of mammary differentiation and tumorigenesis has yet to be determined. Here, using our established mammary-specific Tet2 deletion mouse model, the data reveals that TET2 plays a pivotal role in mammary gland development via directing MaSC/progenitor cell to luminal lineage commitment in vivo. We have also shown that loss of TET2 contributes to impaired luminal lineage commitment, promoted mammary tumor development with deficient ERα expression, and confers tamoxifen resistance in a Tet2 deletion-PyMT breast cancer mouse model.

Unlike TET1 and TET3, TET2 lacks an apparent nuclear localization signal sequence and DNA binding domain^6,7^, while mechanism(s) that mediates recruitment of TET2 to specific chromatin regions remains largely unclear. Our findings here uncover that TET2 interacts with a transcription factor FOXP1 to form a chromatin complex that mediates demethylation of *ESR1, GATA3*, and *FOXA1*, three key genes that are known to coordinately orchestrate luminal lineage specification and endocrine response in the mammary gland^11–15^. It is also reported that these genes are often silenced by DNA methylation in basal-like breast cancers that are highly resistant to anti-estrogen treatments^16,17^. As a result, this study provides a role for TET2 that underlies breast cancer resistance to anti-estrogen treatments, and our in vivo Tet2 deletion breast cancer mouse model will be a valuable tool for studying the associated mechanism(s) for development of anti-cancer therapies.

## Results

### Loss of TET2 leads to impaired luminal lineage commitment

**Fig. 1:**
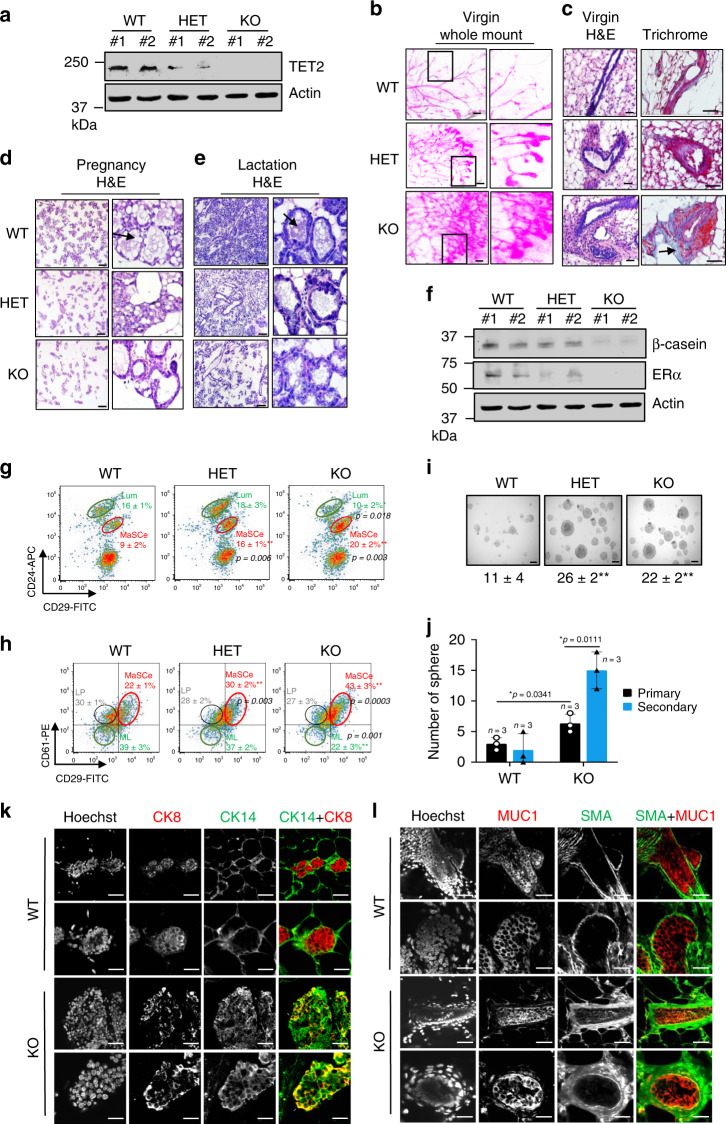
Loss of TET2 leads to dysregulated lobuloalveolar development and impaired luminal lineage commitment. **a** Immunoblot showing TET2 protein expression in WT, HET, and KO mammary tissues. **b**, **c** Whole mount, H&E, and Masson’s Trichrome staining of mammary tissues from 7-week-old WT, HET, and KO virgin female mice. Arrow indicating light blue staining of collagen enriched fibrosis regions (scale bar: 50 μm). **d**, **e** H&E staining of mammary tissues from WT, HET, and KO pregnant mice (day 18.5, scale bar: 50 μm) and lactating mice (day 10, scale bar: 200 μm). Arrows indicating lipid droplets in pregnant mouse tissues (**d**) and milk in lactating mouse tissues (**e**), respectively. **f** Immunoblots showing expression of β-casein and estrogen receptor-α (ERα). **g** Flow cytometry analysis showing the percentage of the basal MaSC-enriched cell population (MaSCe, Lin^−^CD24^+^CD29^hi^, indicated by a red circle) and luminal cell population (Lum, Lin^−^CD24^+^CD29^lo^, indicated by a green circle), and **h** the percentage of basal MaSC-enriched cell population (MaSCe, Lin^−^CD24^+^CD29^hi^CD61^hi^, indicated by a red circle), luminal progenitor cell population (LP, Lin^−^CD24^+^CD29^lo^CD61^hi^, indicated by a black circle), and mature luminal cell population (ML, Lin^−^CD24^+^CD29^lo^CD61^lo^, indicated by a green circle) isolated from *n* = 3 biologically independent mammary epithelial cell samples of 7-week-old- WT, HET, and KO female mice (*n* = 3 animals/group). **i** Representative image and the number of primary mammospheres (scale bar: 50 μm) generated from WT, HET, and KO mammary epithelial cells per 1000 seeding cells (*n* = 3 independent experiments), and **j** the number of the serially passaged spheres generated from WT and KO cells (*n* = 3 independent experiments). Data were presented as mean ± SD. *p*-values were determined by two-sided Student’s *t*-test; asterisk indicates *p* < 0.05, double asterisks indicate *p* < 0.01 (compared to WT). **k**, **l** Representative confocal immunofluorescence images showing co-staining of CK8 with CK14 or MUC1 with SMA in mammary glands from 7-week-old- WT and KO female mice (scale bar: 50 μm). Source data are provided as a source data file.

To determine the physiological role of TET2 signaling in regulation of mammary cell fate in vivo, we have established a mammary specific Tet2 knock-out mouse model. Briefly, Tet2^f/f^ mice possess loxP sites flanking exon 3 (B6;129STet2^tm1.1Iaai/J^, The Jackson Laboratory) were bred with mice that expressed the Cre recombinase under the control of the mouse mammary tumor virus promoter (MMTV-Cre, The Jackson Laboratory) to specifically knock-out Tet2 in the mouse mammary epithelium (Supplementary Fig. 1a, b). TET2 is highly expressed in the mammary luminal cell population (Lum) compared with the basal MaSC-enriched cell population (MaSCe, Supplementary Fig. 1c). Tet2 status and protein expression in MMTV-Cre;Tet2^+/+^ (WT), MMTV-Cre;Tet2^f/+^ (HET), MMTV-Cre;Tet2^f/f^ (KO) mice were verified using PCR genotyping, immunoblotting, and flow cytometry intracellular staining (Fig. 1a, Supplementary Fig. 1a–d). We found that, compared with the 7-week-old virgin WT littermates, mammary glands of the HET and KO mice exhibited abnormal gland development as indicated by enhanced ductal branching, increased number of terminal end bud (TEB), enlarged size of TEB, along with extended fibrosis and hyperplasic lesions (Fig. 1b, c, Supplementary Fig. 1e–h, fibrosis was indicated by trichrome blue collagen staining). Compared with the pregnant and lactating WT littermates, mammary glands of the HET and KO mice exhibited defective luminal-alveolar development, as evidenced by reduced number of lobuloalveoli during pregnancy, where the majority of these alveoli were deficient in lipid droplet-like morphology (lipid droplet-positive alveoli: WT 83% vs. HET 29% vs. KO 13%, Fig. 1d, Supplementary Fig. 1i, j, arrow indicates lipid droplet), and had little milk production during lactation (Fig. 1e, arrow indicates milk), accompanied by repressed protein expression of the luminal cell markers, ERα and β-casein (Fig. 1f), pointing to a defective luminal cell differentiation. To examine whether luminal cell differentiation from MaSC or mammary progenitor cells is indeed impaired by *Tet2* deletion, we analyzed surface lineage markers using flow cytometry to profile the basal MaSC-enriched cell population (MaSCe, Lin^−^CD24^+^CD29^hi^) and the luminal cell population (Lum, Lin^−^CD24^+^CD29^lo^), including the luminal progenitor cells (LP, Lin^−^CD24^+^CD29^lo^CD61^+^), and mature luminal cells (ML, Lin^−^CD24^+^CD29^lo^CD61^−^), isolated from 7-week-old virgin WT, HET, and KO mouse mammary glands. Compared with WT, KO mammary gland had about 2-fold increase in the basal MaSC-enriched cell population (MaSCe, WT 9% vs. KO 20% by Lin^−^CD24^+^CD29^hi^, Fig. 1g; WT 22% vs. KO 43% by Lin^−^CD24^+^CD29^hi^CD61^hi^, Fig. 1h), along with a decrease in the luminal cell population, where the mature luminal cell population was most significantly diminished (ML, WT 39% vs. KO 22%, Fig. 1h). Concordantly, MaSC-enriched cell population isolated from the KO mammary gland was able to form 2-fold more mammospheres than WT cells (WT 11 vs. KO 22, per 1000 seeding cells, Fig. 1i), and was indeed highly enriched in the sphere-forming MaSCs as shown by in vitro limiting dilution analysis (MaSC frequency- WT 1/398 vs. KO 1/112, Supplementary Fig. 1k). We then dissociated the primary spheres into single-cell suspensions and subjected them to secondary sphere cultures. Compared with WT, KO MaSCs showed continuingly elevated sphere formation at each passage (Fig. 1j), suggesting an enhanced self-renewing potential of the MaSCs from the KO mammary gland.

To determine whether luminal cell lineage commitment is indeed affected by Tet2 deletion, we analyzed expression patterns of the luminal cell lineage markers, Cytokeratin 8 (CK8) and Mucin1 (MUC1), along with the basal/myoepithelial cell lineage markers, Cytokeratin 14 (CK14) and α-smooth muscle actin (SMA), in the mammary tissue sections from 7-week-old WT, HET, and KO animals. We found that loss of TET2 led to aberrant lineage commitment switched from a predominantly luminal phenotype (CK8^+^ or MUC1^+^) to a mixed luminal and basal/myoepithelial bi-lineage phenotype (CK8^+^CK14^+^ or MUC1^+^SMA^+^ double-positive staining, Fig. 1k, l). Using three-dimensional acinar differentiation culture of mammary epithelial cells isolated from WT and KO mammary glands, we found that, compared with WT, KO acini displayed disorganized acinar morphology and luminal filling, along with disrupted expression of α6-integrin (basal polarity marker) and E-cadherin (epithelial cell marker), pointing to a dysregulated epithelial cell polarity by *Tet2* deletion (Supplementary Fig. 1l). In the mammary tissue section where WT acini showed an oriented, predominantly luminal phenotype (CK8^+^CK14^−^), KO acini exhibited irregular and mixed luminal and basal/myoepithelial bi-lineage marker expression (CK8^+^CK14^+^ double-positive staining, Supplementary Fig. 1m), pointing to a defective luminal lineage commitment. Our data also showed that the CK8^+^CK14^+^ double-positive bi-lineage cell population, which was known to recapitulate a bi-potent progenitor cell population^18–20^, highly expressed the luminal progenitor cell markers, Prom1 and Nrdg2 (encoding CD133 and NRDG2, respectively) as well as the basal/myoepithelial cell markers, Krt5 and Krt14 (encoding CK5 and CK14, respectively), but it was deficient in the expression of the mature luminal cell marker Krt18 (encoding CK18) as compared with the non-bi-lineage population (Supplementary Fig. 1n). Together, these data suggest that TET2 plays a critical role in normal mammary gland development and luminal cell differentiation.

### TET2–FOXP1 complex mediates luminal cell differentiation

**Fig. 2:**
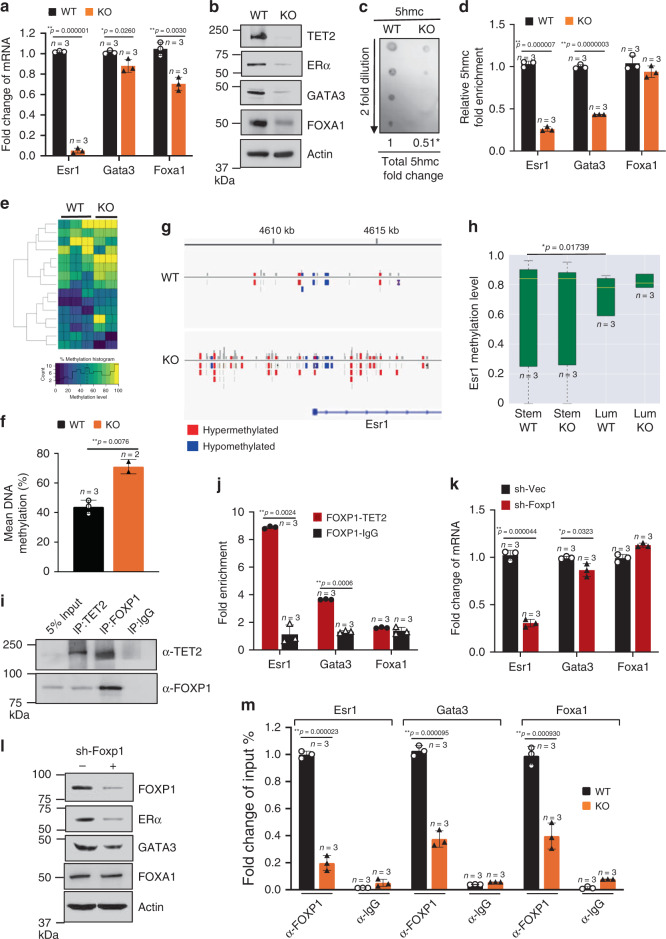
TET2–FOXP1 transcription complex mediates gene expression associated with luminal cell differentiation. **a**, **b** Expression levels of the indicated genes and proteins (ERα, GATA3, FOXA1, *n* = 3 animals/group), **c** dot blot showing total 5hmc levels and fold change of 5hmc (KO vs. WT), **d** fold enrichment of 5hmc in *Esr1*, *Gata3*, and *Foxa1* genes of mammary epithelial cells from 7-week-old WT and KO female mice (*n* = 3 animals/group). **e** Heat map showing visualization and grouping of the samples based on differential DNA methylation, and **f** bar graph showing mean DNA methylation levels across genome from biologically independent mammary epithelial DNA samples of 7-week-old WT and KO female mice (WT *n* = 3, KO *n* = 2 animals). **g** Representative methylation track providing visualization of DNA methylation status surrounding *Esr1* gene of mammary epithelial cells from 7-week-old WT and KO female mice (WT *n* = 3, KO *n* = 2 animals). **h** Distribution of targeted bisulfite methylation level of *Esr1* of sorted MaSC-enriched cells (Stem, Lin^−^CD24^+^CD29^hi^) and Luminal cells (Lum, Lin^−^CD24^+^CD29^lo^) from 7-week-old WT and KO female mice (*n* = 3 animals/group). Boxplot showing the 2nd quartile (median) as a yellow line, and 1st quartile and 3rd quartile as the bottom and upper bounds of the box, respectively. The upper whisker extends from the upper edge of the box to the largest value no further than 1.5*IQR (interquartile range) from the edge. The lower whisker extends from the lower edge of the box to the smallest value at most 1.5*IQR from the edge. **i** Reciprocal co-immunoprecipitation showing endogenous FOXP1 and TET2 interaction in mouse mammary epithelial cells. **j** Bar graph showing fold enrichment of FOXP1–TET2 complex at *Esr1*, *Gata3*, and *Foxa1* regulatory elements using sequential-ChIP-qPCR analysis (n = 3 independent experiments). **k**, **l** Expression of *Esr1*, *Gata3*, and *Foxa1* genes and their protein levels in mammary epithelial cells isolated from 7-week-old WT female mice and stably expressed shFOXP1 or the control vector (*n* = 3 independent experiments). **m** Fold enrichment of FOXP1 at *Esr1*, *Gata3*, and *Foxa1* regulatory elements in WT and KO mammary epithelial cells (*n* = 3 independent experiments). Data were presented as mean ± SD. *p*-values were determined by two-sided Student’s *t*-test; asterisk indicates *p* < 0.05, double asterisks indicate *p* < 0.01. Source data are provided as a source data file.

FOXA1, GATA3, and ESR1 are key transcription factors that coordinately orchestrate luminal lineage specification and endocrine response in the mammary gland^11–15,21^. It is known that these genes are often silenced by DNA methylation in basal-like breast cancers^16,17^, including triple-negative breast cancer (TNBC, negative for ER/PR/HER2 expression) that represents one of the most aggressive types of human cancer and is highly resistant to virtually all targeted therapies and anti-estrogen treatments. Interestingly, we found that compared with WT mammary epithelial cells, *Foxa1, Gata3*, and *Esr1* mRNA and protein expression levels were reduced in HET and KO cells (Fig. 2a, b), where the total 5hmc level along with the 5hmc levels in these genes were downregulated (Fig. 2c, d, Supplementary Fig. 2a). The data revealed that Tet2 haploinsufficiency (HET) is sufficient to confer significant phenotypic changes and the reduced 5hmc level associated with *Esr1* repression in a gene dose-dependent manner (Fig. 1b–f, Supplementary Fig. 2a, HET compared with WT and KO cells). However, Tet2 haploinsufficiency fails to perturb the 5hmc level in *Gata3* gene (Supplementary Fig. 2a, HET compared with WT and KO), suggesting that Tet2 gene dosage effect on DNA demethylation may be context/gene-dependent.

To further determine the effect of TET2 ablation on global DNA methylation, we performed genome-wide bisulfite sequencing analysis (enhanced reduced representation bisulfite sequencing, ERRBS) at the single base pair resolution with a broad coverage within and outside of CpG islands^22^. We found that concordant with the reduced total 5hmc level, loss of TET2 enhanced global DNA methylation level by greater than 1.6-fold (WT 44% vs. KO 71%, Fig. 2e, f). Compared with WT cells, KO cells had more than 2-fold increase of total methylated regions (KO vs. WT: 3580,695 vs. 1541,255 methylated regions, Supplementary Fig. 2b), and that KO cells gained methylation in 54% of the intragenic regions (Supplementary Fig. 2c, including 224 intergenic CpG sites, 108 promoter CpG sites, 71 intron CpG sites, 12 exons CpG sites), where the enhancers and regulatory elements are located. Gene ontology analysis of the annotated gene targets that gained DNA methylation in KO cells revealed that these targets were most significantly enriched for the functional annotation group involved in regulation of neuron differentiation process (Supplementary Data 1), where several of these annotated genes, including *Asap1, Robo2, Nedd4l*, have been known to be involved in negative regulation of mammary stem cell and stem cell differentiation^23–25^ (Supplementary Data 1).

Consistent with the reduced mRNA and protein expression levels in KO cells (Fig. 2a, b), *Esr1, Gata3*, and *Foxa1* genes were hypermethylated in the regulatory elements of KO cells (Fig. 2g, Supplementary Fig. 2d). Targeted bisulfate sequencing result further revealed that compared with MaSC, methylation of *Esr1* gene was significantly lowered in the luminal cells, while deletion of *Tet2* could increase DNA methylation level of *Esr1* in the luminal cell population to a level similar to that in the stem cell population (Fig. 2h). Together, these data suggest that loss of TET2 expression contributes to DNA methylation and repression of the three key genes that have been associated with luminal cell differentiation in the mammary gland, namely *Esr1, Gata3*, and *Foxa1*.

Unlike other TET proteins, TET2 does not have a putative DNA-binding domain^6^ and can be recruited to specific chromatin regions through interaction with DNA-binding transcription factors^10,26–28^. To identify key transcription regulatory elements and putative transcription factors that may serve as co-activators of TET2 to mediate gene expression of *Foxa1, Gata3*, and *Esr1*, we performed promoter analysis (Genomatix MetInspector) and found that Forkhead box protein P1 (FOXP1) binding motif was the most enriched in the promoter regions of all three FOXA1, GATA3, and ESR1 genes (high matrix consensus score greater than 0.99) and might serve as a potential transcription factor to modulate expression of these genes (Supplementary Data 2). Recent report has demonstrated that FOXP1 is a crucial transcription factor for orchestrating mouse MaSC differentiation and mammary gland development^29^. We performed a global motif analysis of the differentially methylated DNA sequences and revealed that a transcription factor binding motif (TGTTTAC) shared by the forkhead-box transcription factor family, including FOXP1, was highly enriched in the regions that gained DNA methylation in KO cells (Supplementary Data 3). We further found that FOXP1 protein was highly associated with TET2 protein as endogenous TET2 protein could be reciprocally co-immunoprecipitated with endogenous FOXP1 protein in WT mouse mammary epithelial cells and also in human mammary epithelial cells, MCF12A (Fig. 2i, Supplementary Fig. 2e). Using sequential chromatin immunoprecipitation (ChIP) assay, we showed that TET2–FOXP1 complex co-occupied in regulatory elements of *Gata3*, *Foxa1,* and *Esr1* genes (Fig. 2j). We showed that FOXP1 could bind to *Esr1* genes via putative FOXP1 binding motifs in the promoter region (binding sites at 198 bp through 996 bp upstream TSS) and also in the enhancer region (binding sites at 3160 bp through 3653 bp upstream TSS), where FOXP1–TET2 mainly co-occupied in the GC-rich enhancer region (Supplementary Fig. 2f, g). Knockdown of FOXP1 decreased gene expression of *Gata3* and *Esr1*, along with diminishing ERα protein expression in WT mouse mammary epithelial cells as well as in human breast cancer cells, MCF7 (Fig. 2k,l, Supplementary Fig. 2h). It was shown that TET2 binding to enhancers played a pivotal role for chromatin accessibility and recruitment of transcription factors^30^. Concordantly, we showed that deletion of *Tet2* abolished FOXP1 recruitment to the *Esr1, Gata3* and *Foxa1* genes (Fig. 2m, Supplementary Fig. 2i). Together, these data suggest that FOXP1 directs TET2 binding to specific genes (*Esr1, Gata3*, and *Foxa1*), where TET2 is required for FOXP1 chromatin recruitment and coordinately mediates expression of the genes involved in luminal cell differentiation.

### Loss of TET2 expression confers endocrine resistance

**Fig. 3:**
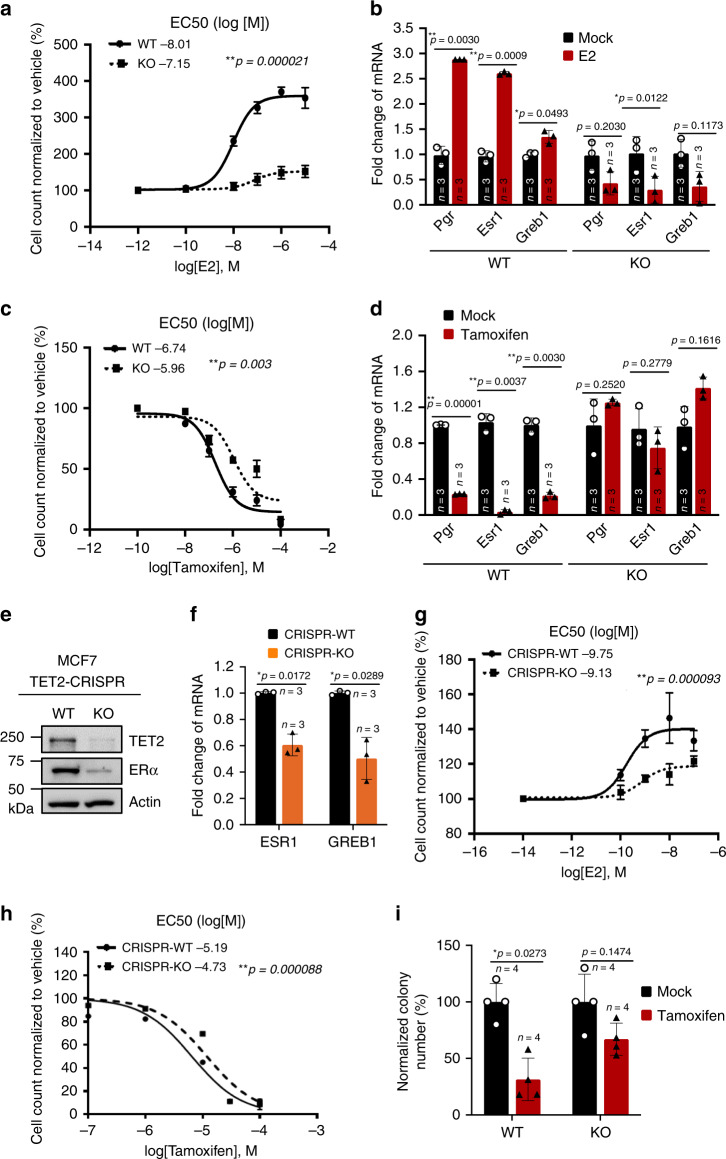
Loss of TET2 expression confers endocrine resistance in vitro. **a** The dose–response curve showing the normalized percentage of surviving cells with EC50 (*n* = 3 independent experiments), and **b** *Esr1*, *Pgr*, and *Greb1* expression levels of mammary epithelial cells isolated from 7-week-old WT and KO female mice and treated with Estradiol (E2, 10^−7^ M) or the control vehicle for 72 h (*n* = 3 independent experiments). **c** The dose–response curve showing the normalized percentage of surviving cells with EC50 (*n* = 3 independent experiments), and **d** *Esr1*, *Pgr*, and *Greb1* expression levels of mammary epithelial cells isolated from 7-week-old WT and KO female mice and treated with tamoxifen (10^−7^ M) or control vehicle for 24 h (*n* = 3 independent experiments). **e** Protein expression of TET2 and ERα, and **f** *ESR1* and *GREB1* gene expression levels in MCF7 cells stably expressing CRISPR-TET2 (CRISPR-KO) or vector (CRISPR-WT) (*n* = 3 independent experiments). **g**, **h** The dose–response curve showing the normalized percentage of surviving cells with EC50 of CRISPR-WT- and CRISPR-KO-MCF7 cells treated with **g** Estradiol (E2) or control vehicle (*n* = 3 independent experiments), **h** Tamoxifen or control vehicle in phenol red free charcoal stripped FBS media for 72 h (*n* = 3 independent experiments). **i** The normalized percentage of colony formation of CRISPR-WT- and CRISPR-KO-MCF7 cells treated with tamoxifen (5 × 10^−6^ M) or control vehicle (*n* = 4 independent experiments). Data were presented as mean ± SD. *p*-values were determined by two-sided Student’s *t*-test; asterisk indicates *p* < 0.05, double asterisks indicate *p* < 0.01. Source data are provided as a source data file.

Since deletion of TET2 leads to impaired ERα expression (Fig. 2b), we next asked whether loss of TET2 expression conferred endocrine resistance in mammary epithelial cells and breast cancer cells. Primary mammary epithelial cells isolated from WT and KO mouse mammary glands were treated with various concentrations of Estrogen (Estradiol, E2) and tamoxifen, one of the most commonly used selective ER modulators (SERM) for breast cancer hormone therapy^31^. We found that estrogen-dependent cell growth and estrogen-induced ER target gene expression (*Pgr, Esr1, Greb1*) were abrogated upon Tet2 deletion (Fig. 3a, b). Similarly, compared with WT mammary epithelial cells, loss of TET2 in KO cells resulted in resistance to tamoxifen-mediated cell growth inhibition as evidenced by a an elevated EC_50_ (10^−6.74^ M vs. 10^−5.96^ M, Fig. 3c), accompanied by a diminished sensitivity to tamoxifen-mediated suppression of ER target gene expression (Fig. 3d).

To further determine the role of TET2 in conferring endocrine resistance in human breast cancer cells, we generated a stable TET2 knock-out cell line using the CRISPR-Cas9 system in ERα-positive, luminal breast cancer MCF7 cells. Consistent with the results from mouse mammary epithelial cells, loss of TET2 led to decreased expression of ERα and ERα target gene, GREB1 (Fig. 3e, f). We found that compared with TET2-WT-MCF7 cells, TET2-KO-MCF7 cells exhibited a declined sensitivity to estrogen-induced cell growth (EC_50_: 10^−9.75^ M vs. 10^−9.13^ M, Fig. 3g), and also developed resistance to tamoxifen-mediated cell growth inhibition (EC_50_: 10^−5.19^ M vs. 10^−4.73^ M, Fig. 3h), as well as resistance to colony formation inhibition (Fig. 3i). Together these data suggest that loss of TET2 contributes to loss of ERα expression and defective ER signaling that confers endocrine resistance in mammary epithelial cells and breast cancer cells.

### Loss of TET2 expression promotes mammary tumor development

**Fig. 4:**
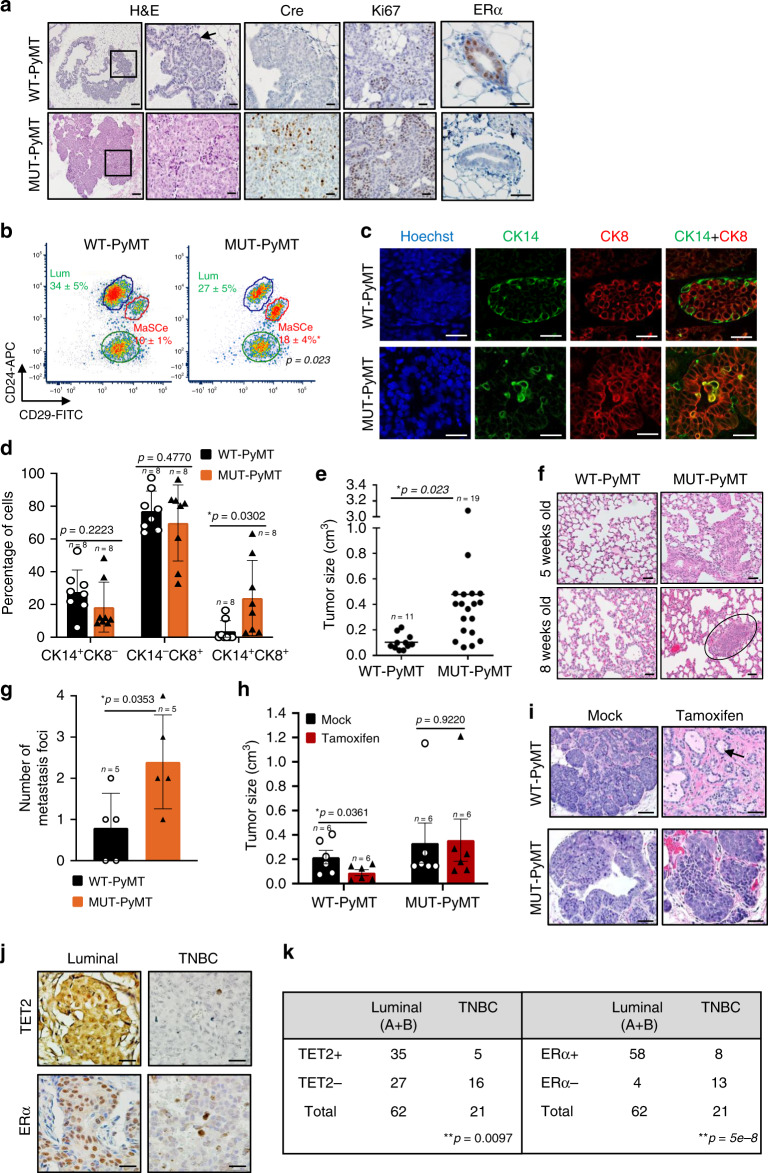
Loss of TET2 expression promotes tumorigenesis and tamoxifen resistance in vivo. **a** Representative immunostaining images of H&E, Cre, Ki67, and ERα in mammary glands of 5-week-old Tet2^f/+^;PyMT (WT-PyMT) and MMTV-Cre;Tet2^f/+^;PyMT (MUT-PyMT) female mice. Arrow indicating premalignant hyperplasia morphology (scale bar: 50 μm). **b** Flow cytometry analysis showing the percentage of basal MaSC-enriched population (MaSCe, Lin^-^CD24^+^CD29^hi^, indicated by a red circle and luminal cell population (Lum, Lin^−^CD24^+^CD29^lo^, indicated by a blue circle) isolated from 6-week-old WT-PyMT and MUT-PyMT female mice (*n* = 3 animals/group). **c** Representative immunofluorescence images showing co-staining of CK8 (red) and CK14 (green) in mammary glands of 7-week-old WT-PyMT and MUT-PyMT female mice (scale bar: 50 μm), and **d** bar graphs showing percentage of CK14^+^CK8^−^, CK14^−^CK8^+^, and CK14^+^CK8^+^cells. *n* = 8 data points analyzed from eight independent tissue section staining images of two animals for each group. Data were presented as mean ± SD. *p*-values were determined by two-sided Student’s *t*-test between the indicated two groups; asterisk indicates *p* < 0.05. **e** Tumor size of all the tumors collected from WT-PyMT and MUT-PyMT female mice at 12–13 weeks of age. *n* = 11 WT-PyMT tumors and *n* = 19 MUT-PyMT tumors were collected from three animals for each group. Data were presented as mean ± SD. *p*-values were determined by two-sided Student’s *t*-test between the indicated two groups; asterisk indicates *p* < 0.05. **f** Lung tissues from WT-PyMT and MUT-PyMT female mice at 5 weeks and 8 weeks of age (scale bar: 50 μm). The black ellipse indicating metastasis foci. **g** Bar graph showing number of histologically identified metastasis loci per lung/animal (*n* = 5 animals/group). Data were presented as mean ± SD. *p*-values were determined by two-sided Student’s *t*-test between the indicated two groups; asterisk indicates *p* < 0.05. **h**, **i** Tumor size and tumor phenotype of 7-week-old WT-PyMT and MUT-PyMT female mice that had been treated with mock control vehicle, corn oil, or tamoxifen (i.p. 25 mg/kg) daily for 5 consecutive days per week for 4 weeks (*n* = 6 animals/group). Arrow indicating normal single-layered ductal epithelial morphology (scale bar: 100 μm). Data were presented as mean ± SD. *p*-values were determined by two-sided Student’s *t*-test; asterisk indicates *p* < 0.05. **j** Representative immunostaining images of TET2 and ERα, and **k** Chi-Square analysis of TET2 and ERα expression in 83 human breast tumor specimens of luminal breast cancers and triple negative breast cancers. (−): negative/low staining, (+): positive/high staining (scale bar: 50 μm). Asterisk indicates *p* < 0.05; double asterisks indicate *p* < 0.01. Source data are provided as a source data file.

To further determine the role of TET2 in breast tumorigenesis, we generated a Tet2-deletion breast cancer mouse model by breeding our Tet2-deletion mouse model (MMTV-Cre;Tet2^f/+^) with an established breast cancer mouse model, MMTV-PyMT (PyMT). PyMT mouse expresses polyoma middle T (PyMT) oncogenic protein in mouse mammary epithelium and develops spontaneous luminal-like ER-positive premalignant mammary lesions and adenoma at 4–6 weeks of age, which further progresses to ER-negative mammary carcinoma around 8–12 weeks of age, along with spontaneous lung metastases by 12 weeks of age in PyMT mice^32^. We collected the mammary glands from Tet2^f/+^;PyMT (WT-PyMT) and MMTV-Cre;Tet2^f/+^;PyMT (MUT-PyMT) at 5 weeks of age to verify the effects of *Tet2* deletion on the early onset of mammary tumor development. The data revealed that MUT-PyMT accelerated development of highly proliferative mammary carcinoma at 5 weeks of age when WT-PyMT had only pre-malignant lesions (Fig. 4a, Supplementary Fig. 3a–c, tumor-free survival: WT-PyMT 8 weeks vs. MUT-PyMT 5 weeks; Ki67-positive cells: WT-PyMT 3.7% vs. MUT-PyMT 11.7%). Compared with WT-PyMT, *Tet2* deletion led to lost ERα expression in MUT-PyMT mammary glands (Fig. 4a, Supplementary Fig. 3d, ERα-positive cells: WT-PyMT 10.8% vs. MUT-PyMT 1.8%). Loss of TET2 also increased the basal MaSC-enriched cell population (Fig. 4b, MaSCe, WT-PyMT 10% to MUT-PyMT 18%) and resulted in aberrant lineage commitment with a mixed bi-lineage phenotype (Fig. 4c, d, CK8^+^CK14^+^ double-positively stained cells: WT-PyMT 3.7% vs. MUT-PyMT 24%).

Furthermore, we found that compared with WT-PyMT, MUT-PyMT mice generated 4.8-fold larger mammary tumors with a more aggressive tumor phenotype (Fig. 4a, c, e, tumor size: WT-PyMT 0.1 cm^3^ vs. MUT-PyMT 0.48 cm^3^, *n* = 3 animals/group). MUT-PyMT mice also had accelerated development of spontaneous lung lesions at 5 weeks of age, followed by the development of 3-fold more lung micrometastases at 8 weeks of age as compared to WT-PyMT mice (Fig. 4f, g, number of metastasis foci: WT-PyMT 0.8 vs. MUT-PyMT 2.4 foci per lung/animal, *n* = 5 animals/group).

### Loss of TET2 expression confers tamoxifen resistance in vivo

Since ERα expression was deficient in MUT-PyMT mammary glands, we examined whether loss of TET2 would confer tamoxifen resistence in vivo by injecting WT-PyMT and MUT-PyMT mice intraperitoneally with mock control vehicle, corn oil, or Tamoxifen (25 mg/kg daily, five consecutive days per week for 4 weeks, *n* = 6 animals/group). Compared with the control group, we found that tamoxifen was able to reduce 60% of WT-PyMT tumor growth (Fig. 4h, tumor size: WT-PyMT 0.22 cm^3^ vs. MUT-PyMT 0.09 cm^3^) and converted an aggressive tumor phenotype to a normal ductal epithelial morphology (single-layered epithelium), mimicking a mammary involution phenotype induced by tamoxifen (Fig. 4i). However, MUT-PyMT tumors utterly failed to respond to tamoxifen treatment (Fig. 4h, i, tumor size: WT-PyMT 0.33 cm^3^ vs. MUT-PyMT 0.36 cm^3^). We have also shown that after tamoxifen treatment, the residual WT-PyMT tumor cells become highly enriched in CK14 expression with modestly diminished CK8 expression (Supplementary Fig. 3e, mock vs. tamoxifen: 1 vs. 7.6- fold (CK14), 1 vs. 0.6- fold (CK8)), suggesting that tamoxifen likely targets CK14-negative luminal cells, and that CK14-positive cells, including the CK14^+^CK8^+^ bi-lineage progenitor cell population and the CK14^+^CK8^−^basal/myoepithelial cell population, are likely resistant to tamoxifen. It has been reported that the bi-lineage cell population manifests progenitor-associated traits and is associated with increased tumorigenicity and poor differentiation state in basal-like breast cancers known to be endocrine resistant^19,20^. Therefore, the enhanced CK14^+^CK8^+^ bi-lineage progenitor cell population in MUT-PyMT mammary tissue may account for the poorly differentiated, tamoxifen resistant MUT-PyMT tumor phenotype.

Consistently, using a cohort of breast cancer patient specimens, we showed that, compared with luminal breast cancers, where TET2 and ERα were predominantly expressed, TNBC exhibited deficient expression of both TET2 and ERα (Fig. 4j, k, *n* = 83, TE2: *p* = 0.0097; ERαː *p* = 5e−8). Together, these data suggest that loss of TET2 promotes and accelerates mammary tumor development, enhances the frequency of lung metastasis with early onset, and also confers intrinsic tamoxifen resistance in the tumor cells, where the TET2 deficiency phenotype is associated with basal-like human breast cancers that are highly resistant to hormone therapy.

## Discussion

It has been shown that TET2 expression is often silenced post-transcriptionally in human cancers, such as by mircroRNA-mediated gene silencing. Previous studies have revealed that TET2 can be a direct target of the Let-7adf cluster in LPS-activated macrophages^33^, it can be suppressed by miR-29 in prostate cancer cells^9^, and it is down-regulated by miR-22 in breast cancer cells^34^. miR-22, a microRNA that is associated with oncogenic signaling and overexpressed in high-grade breast tumors with poor clinical outcomes^26,34,35^, directly targets TET family members, including TET2, which in turn leads to hypermethylation of the mir-200 promoter and induction of breast cancer stemness phenotype and metastasis^34^. The associated TET2 regulatory mechanism was further elaborated by another study that showed TET2 could complex with RARβ to epigenetically activate a cohort of gene targets involved in cell differentiation, including *RUNX1, BMP6, IKZF1* and *CAV1*, and *Mir-200c*^10^. TET2-activated miR-200c in turn targets and suppresses the cell polarity protein PKCζ to promote symmetric cell division of a breast cancer stem cell-like population and direct breast cancer stem cell to the differentiation state in vitro^10^. Here we have further identified that TET2 and FOXP1 form a chromatin complex that mediates demethylation of *ESR1, GATA3*, and *FOXA1*, three key genes that are known to coordinately orchestrate luminal lineage specification in the mammary gland^11–15,36^. The previous studies, together with our findings using the established mammary-specific Tet2 deletion mouse models, demonstrate that through coordinately modulating the gene expression network, TET2 can play a dual role in regulation of MaSCs/progenitor cells and in directing luminal lineage commitment, which is critical for the maintenance of tissue homeostasis and endocrine sensitivity in the mammary gland. Loss of TET2 on the one hand impairs luminal and lobuloalveolar differentiation, as TET2 deficiency significantly diminishes the expression of *Gata3* and *Esr1* genes, which are required for the development of lobuloalveolar structure during pregnancy^37^ (Figs. 1 and 2); on the other hand, loss of TET2 leads to an increased MaSCs/progenitor cells that promote the development of mammary ductal branching network during puberty (Fig. 1). Furthermore, Tet2 ablation-mediated accumulation MaSCs and bi-lineage progenitor cells are associated with poor differentiation tumor phenotype and endocrine resistance^19,20^ (Fig. 4 and Supplementary Fig. 3e, f), which potentially underlies the development of intrinsic resistance to anti-estrogen treatments in aggressive breast cancer.

Unlike TET1 and TET3, which contain CXXC DNA binding domain, TET2 binds to DNA through tissue-specific DNA-binding factors^10,27,38–40^ to activate transcription of respective target genes. Wang et al. has recently reported that TET2 coordinates with MLL3 at enhancers to facilitate the recruitment of transcription factors^26^. It has also been implicated that TET2 can function independently of its catalytic activity though binding to specific enhancers and facilitate transcription factor recruitment^30,41^; as a result, differential stoichiometry between TET2, target enhancers/binding regions, and the associated transcription factors may contribute to the context-dependent TET2 functioning. In our current study, we have shown that TET2 can be guided by its interaction with FOXP1 to specifically bind to and activate the target genes involved in luminal cell differentiation; FOXP1 also likely relies on TET2-mediated demethylation to gain access to chromatin^30^ for transcriptional activation of these target genes. FOXP1 is crucial for orchestrating mouse MaSC differentiation and mammary development^29^. FOXP1 expression is also positively correlated with hormone receptor status and breast cancer sensitivity to endocrine therapy^42,43^. These evidence provide important clinical relevance of TET2-FOXP1 axis to ER signaling regulation and hormone therapy sensitivity in human breast cancers.

ER-positive breast cancers consist about 80% of breast cancers. Despite the benefit of tamoxifen treatment in ER positive breast cancer, around 50% of the patients treated with adjuvant tamoxifen treatment would eventually relapse^17,44^. Resistance developed for treatment may either be *intrinsic*, which is present before the start of any treatment, or the resistance is *acquired* during the course of treatment. On the one hand, ER expression often continues to be expressed in the majority of acquired endocrine-resistant tumors, while *ESR1* mutation occurs in 20% acquired aromatase inhibitor-resistant tumors, and there are other growth factor signaling pathways, including EGFR, HER2, insulin/IGFs, P13K/AKT/mTOR, MAPK, and FGFR, that have been reported as potential mechanisms of acquired endocrine resistance^44^. On the other hand, loss of ER is observed in ~15–20% of *intrinsic* endocrine-resistant breast cancers that exhibit resistance to tamoxifen and other anti-estrogens^45^. Epigenetic silencing of ER due to hypermethylation of ER gene has been reported both in vitro and in vivo^46^. It has been shown that endogenous ER expression can be restored a TNBC cell line (MDA-MB-231) by the treatment of demethylating agent, 5-aza-2′-deoxycytidine, and thereby re-sensitizes TNBC cells to tamoxifen mediated growth inhibition^47^. Our findings have revealed that loss of TET2 accounts for epigenetic silencing of ER that contributes to intrinsic resistance to tamoxifen. Deficiency in TET2 expression/activity may be used as a potential biomarker to predict tamoxifen resistance in human breast cancer. Restoration of TET2 expression/activity is expected to provide therapeutic options for the cohort of breast cancer patients with intrinsic endocrine resistance.

## Methods

### Mice

Tet2^f/f^ mice (B6;129STet2^tm1.1Iaai^, 017375, The Jackson Laboratory) which possess loxP site flanking axon 3 in Tet2 gene were crossed with MMTV-Cre transgenic mice (Tg(MMTV-cre)4Mam/J, 003553, The Jackson Laboratory) that expresses Cre recombinase under the control of mouse mammary tumor virus (MMTV) long terminal repeat (LTR) promoter to give rise to: MMTV-Cre;Tet2^+/+^ (WT), MMTV-Cre;Tet2^f/+^ (HET), MMTV-Cre;Tet2^f/f^ (KO) mice. To generate Te2 deletion breast cancer mouse model, the MMTV-Cre;Tet2^f/+^ mouse line were crossed with the MMTV-PyMT transgenic mouse line (FVB/N-Tg(MMTV-PyVT)634Mul/J, 002374, The Jackson Laboratory) to obtain MMTV-Cre;Tet2^f/+^;PyMT. Mice were housed in a normal pathogen-free environment (12 light/12 dark cycle, 68–72 °F) with access to standard mouse diet and water ad libitum. The virgin female mice in comparison comprised age-matched littermates that were housed in the same cage in synchronized estrus stage^48^ confirmed by visually evaluating the vaginal openings^49^. Seven-week-old Tet2^f/+^;PyMT and MMTV-Cre;Tet2^f/+^;PyMT female mice were treated with corn oil or tamoxifen (i.p. 25 mg/kg, *n* = 6 animals/group) for five consecutive days per week for four weeks. At the end of the treatment, tumors were measured by caliper and tumor volume was calculated using (tumor length × tumor width^2^)/2 and then collected and fixed in 10% neutral formalin for histological evaluation. Experiments were conducted with approval of the Institutional Biosafety Committees and the Animal Care and Use Committees at Purdue University and Roswell Park Comprehensive Cancer Center.

### Dissociation of mouse mammary epithelial cells

Mouse mammary glands were minced and digested for 16 h at 37 °C in complete EpiCult-B medium (5% FBS, 50 μg/mL gentamycin, and supplemented with gentle collagenase/hyaluronidase, Stem Cell Technologies). After lysis of red blood cells in the mixture of Hank’s Balanced Salt Solution (HBSS) with 2% FBS and NH_4_Cl (1:4), a single-cell suspension was obtained by sequential dissociation of the fragments with prewarmed 0.25% trypsin-EDTA for 3 min, followed by prewarmed 5 mg/mL dispase II plus 0.1 mg/mL DNase I for 1 min, and filtration through 40 μm cell strainer. Mouse mammary epithelial cells were grown in 24-well plates (5 × 10^4^ cells per well) with serum free complete EpiCult™-B mouse media following the manufacturer’s instructions (Stem Cell Technologies) and then subjected to the indicated treatment.

### Generation of stable cell lines

The immortalized normal mammary epithelial cell line, MCF12A, and the luminal breast cancer cell line, MCF7, as well as 293T cells, were purchased from American Type Culture Collection (ATCC). MCF12A cells were grown in DMEM-F12 medium supplemented with 5% horse serum, epidermal growth factor (20 ng/ml), insulin (10 ng/ml), cholera toxin (100 ng/ml), hydrocortisone (500 ng/ml) and gentamycin (Sigma). MCF7 cells were cultured with DMEM medium supplemented with 10% FBS, penicillin (50 U/ml), and streptomycin (50 U/ml). To establish stable cell lines, shRNA plasmids (targeting human or mouse FOXP1, Sigma) or CRISPR plasmids (pLV-CRISPR-hTET2, Vector Builder) were co-transfected with lentiviral packaging plasmids (pPAX2 and pMD2.G) into 293T cells. After 48 h incubation, lentiviruses were harvested and used to infect the target cells. Stable cell lines were selected with puromycin (2 μg/mL) treatment for 7 days.

### Whole mount staining of mouse mammary tissues

Entire inguinal mammary gland was removed and directly spread on the glass slides. The tissue fixed in the Carnoy’s solution (mixture of ethanol, chloroform and glacial acetic acid; 6:3:1 ratio) for 4 h at room temperature. Then tissue was gradually hydrated and stained in carmine alum (#07070, Stem Cell Technologies) stain overnight at room temperature. Whole mount was dehydrated in series of ethanol, 50%, 70%, 95 and 100% for 5 min each, cleared in Xylene overnight and kept in methyl salicylate until taking images. Images were taken by using SteREO Discovery V12 microscope.

### Immunohistochemistry staining of mammary tissues

Mouse mammary gland was fixed in 10% buffered formalin and processed as hematoxylin and eosin stained slides and unstained formalin fixed paraffin embedded mammary tissue section slides. Collagen was stained using Trichrome Stain Kit according to the manufacturer’s protocol (TRM-1-IFU, ScyTek). Sections of formalin fixed paraffin embedded mouse mammary gland and human breast cancer tissue microarray slides (BRC1501, Pantomics) were deparaffinized and rehydrated. After heat-induced antigen retrieval, sections were incubated with 5% BSA and then incubated with primary antibodies: anti-TET2 (#ABE364, Millipore Sigma, 1:250), anti-Cre (#15036, Cell Signaling, 1:200), anit-Ki67 (#PIPA519462, Invitrogen, 1:200), and anti-ERα (#ab32063, Abcam, 1:100) overnight at 4 °C. Next, appropriate secondary antibody was applied to the section and visualized with DAB chromogen kit (BioCare Medical). Slides were counterstained with hematoxylin and the images were taken by Olympus BX53 Upright Microscope. For human breast cancer tissue microarray, the histological grading and pathological annotation (tumor grade and subtype) were provided by Pantomics. The correlation between the expression levels of the proteins and with the tumor grade was analyzed using Chi-Square test.

### Immunofluorescence staining of mouse mammary tissues

The fresh mouse mammary gland was sliced and fixed in 4% paraformaldehyde for one hour. The tissue slices were incubated with primary antibodies, including anti-CK8 (#ab59400, Abcam, 1:250), anti-CK14 (#ab7800, Abcam, 1:250), anti-MUC1 (#ab45167, Abcam, 1:250), and anti-α-SMA (#A5228, Sigma Aldrich, 1:500), in PBS containing 0.5% Triton X-100 and 5% BSA overnight at 4 °C. The specimens were washed with PBS with 0.1% tween 20 three times and incubated with a fluorochrome-conjugated second antibodies, including Rhodamine Red-conjugated goat anti rabbit IgG (#111-295-003, Jackson Immunoresearch, 1:400) and FITC-conjugated goat anti mouse IgG (#115-095-003, Jackson Immunoresearch, 1:400), overnight at 4 °C. After washing, the samples were incubated with Hoechst (#H3570, Life technology, 1:1000) for 10 min at room temperature. Tissue samples were placed on glass slides and mounted with mounting solution. After cover slipping, images were taken by Olympus FV10i-LIV Laser Scanning Microscope (Fluoview v3.0).

### Flow cytometry analysis

Cells at a concentration of 1 × 10^6^ per 100 µl of staining buffer (BD Biosciences) were incubated on ice for 30 min with the following antibodies: FITC-conjugated anti-CD29 (#561796, BD Biosciences, 1:100), PE-Cy7-conjugated CD29 (#25029182, eBioscience, 1:100), PE-conjugated anti-CD29 (#25029180, eBioscience, 1:100), APC-conjugated anti-CD24 (#562349, BD Biosciences, 1:100), PE-conjugate anti-CD24 (#553262, BD Biosciences, 1:100), PE-conjugated anti-CD61 (#561910, BD Biosciences, 1:100), PerCP-Cy™5.5 Mouse Lineage Antibody Cocktail (#561317, BD Biosciences, 1:100), anti-TET2 (#36449, Cell Signaling Technology, 1:100), and Alexa Fluor® 488-conjugated Anti-rabbit IgG (H + L) F(ab’)2 Fragment (#A-11070, ThermoFisher Scientific, 1:500). Stained cells were subjected to BD Canto II analysis (BD FACS Diva 8), and flow cytometry data (mean% ± SD) was analyzed by FCS express 6 (Denovo Software) from three independent experiments with gating boundaries determined by using antibody isotype controls. FACS sorted cells were fixed and permeabilized, stained with Alexa Fluor® 594-conjugated Cytokeratin 8 Antibody (#NB120-9287AF594, Novus Biologicals, 1:200), Alexa Fluor® 647-conjugated Cytokeratin 14 Antibody (#NBP2-47720AF647, Novus Biologicals, 1:200)^19,20^, and then subjected to BD FACSAria FACS sorting followed by standard qRT-PCR analysis.

### Dose–response curve and EC50 calculation

Data points of the dose–response curve were based on the surviving cell number using MTT assay counted in three independent experiments. EC50 value for each group were calculated by fitting the data points to the four-parameter logistic sigmoidal dose–response curve: where X is the logarithm of concentration and Y is the normalized cell number counts (%). Curve fitting was performed with GraphPad Prism 8 (GraphPad Software, Inc.).

### Immunoblotting and immunoprecipitation assay

Immunoblotting and immunoprecipitation were performed according to standard protocol with the following antibodies: anti-TET2 (#36449, Cell Signaling Technology; #61389, Active Motif, 1:1000), anti-β-Actin (#A5316, Sigma, 1:5000), anti-β-Casein (#sc166530, Santa Cruz, 1:1000), anti-ERα (#ab32063, Abcam, 1:1000), anti-GATA3 (#PA520892, Thermo Fisher Scientific, 1:1000), anti-FOXA1 (sc101058, Santa Cruz, 1:1000), and anti-FOXP1 (#4402T, Cell Signaling Technologies, 1:1000). HRP-conjugated secondary antibody (#610-103-121 and #610-103-122, Rockland Immunochemicals, 1:5000).

### Genome-wide bisulfite sequencing and data analysis

Enhanced reduced representation bisulfite sequencing (ERRBS), an enhanced bisulfite-based sequencing to detect methylation covering nearly all CpG islands, gene promoters, genetic regulatory elements, gene bodies, and repeated DNA sequences, was performed by Epigentek. DNA was isolated from cells from WT and KO mouse mammary glands and concentration was measured with Picogreen fluorescence method. The samples were subjected to enzymatic digestion (MSP1 + TaqI), library preparation, bisulfite conversion (Methylamp DNA Bisulfite Conversion Kit, Epigentek), Bioanalyzer QC, KAPA library quantification, and multiplex next-generation sequencing on an Illumina HiSeq4000. Quality control was performed on the Illumina raw reads using FASTQC (version 0.11.8). Quality and adapter trimming was performed on the raw reads using Trim Galore (version 0.5.0). Trimmed reads were mapped to the UCSC mus musculus GRCm38 genome sequence using Bismark (version 0.203.0). DMC (differentially methylated cytosines) analysis was performed in the CpG context. Samples were filtered by coverage (minimum 5), normalized, merged, and subjected to DMC identification. The identified DMCs were annotated against the RefSeq genes and the CpG islands/shores. Read counts with methylated cytosines and unmethylated cytosines in each region were summed up. Percentage of methylation is calculated as (#methylated cytosines)/(#methylated cytosines + #unmethylated cytosines) * 100. For each sample, Bismark generated coverage files were used to extract methylation percentages for common genomic loci among all samples. These percentages were then supplied as a dataframe in R to generate the filtered and non-filtered heatmaps. Bisulfite methylation track graph was generated by Integrative Genomics Viewer (IGV, Broad Institute, version 2.8.0)) according to the instructions (https://software.broadinstitute.org/software/igv/UserGuide). Gene ID’s of differentially methylated regions as determined by methylKit were extracted and used as input for gene ontology enrichment analysis and motif enrichment analysis using Homer (version sv4.11.1).

### Targeted bisulfite sequencing and data analysis

Isolated total mammary epithelial cells, including luminal and myoepithelial cells, from WT and KO animals were stained with Zombie Violet Fixable Viability Kit (#423113, Biolegend). After washing, cells were stained with 1:100 FITC-conjugated anti-CD29 (#561796, BD Biosciences, 1:100), APC-conjugated anti-CD24 (#562349, BD Biosciences, 1:100), and PerCP-Cy5.5 Mouse Lineage Antibody Cocktail (#561317, BD Biosciences, 1:100). Stained cells were sorted by BD FACS Aria Fusion for basal MaSC enriched cell population (MaSCe, Lin^−^CD24^+^CD29^hi^) and luminal cell population (Lum, Lin^−^CD24^+^CD29^lo^). Genomic DNA was extracted from each cell population using Blood & Cell Culture DNA Mini Kit (#13323, Qiagen) according to the manufacturer’s instruction; Three primers were designed in the CpG regions of ERα (chr10: 4609801-4610189) and targeted bisulfite sequencing was performed by Zymo Research. Briefly, assays were designed targeting CpG sites in the specified regions of interest (ROI) using primers created with DNA-specific primer design tool, Rosefinch (Zymo Research). Samples were bisulfite converted using the EZ DNA Methylation-LightningTM Kit (Zymo Research) according to the manufacturer’s instructions. Multiplex amplification of all samples using ROI specific primer pairs. The resulting amplicons were pooled for harvesting and subsequent barcoding according to the Fluidigm instrument’s guidelines. Samples were then prepared for parallel sequencing using a MiSeq V2 300 bp Reagent Kit and paired-end sequencing protocol according to the manufacturer’s guidelines. Sequence reads of each sample were identified using standard Illumina methylation calling software (Bismark Bowtie2). The boxplot displays the median DNA methylation levels as well as the distribution of methylation levels within a sample. The boxplots show the 2nd quartile (median) as a yellow line, and 1st quartile and 3rd quartile as the bottom and upper bounds of the box, respectively. The upper whisker extends from the upper edge of the box to the largest value no further than 1.5*IQR (or interquartile range) from the edge. The lower whisker extends from the lower edge of the box to the smallest value at most 1.5*IQR from the edge.

### Chromatin immunoprecipitation and real-time PCR

The sequences of Esr1, Gata3, and Foxa1 promoters were obtained from UCSC Genome Database. Analysis of putative transcription factor binding sites on Esr1, Gata3 and Foxa1 promoter was done by TRED (Cold Spring Harbor Laboratory) and MatInspector (Genomatix). ChIP experiment was modified from the EZ-CHIP (EMD Millipore/Upstate) protocol. The cells and minced tissues were fixed in 1% formaldehyde for 10 min at room temperature under rotation, followed by quenching in 0.125 M glycine for 5 min. The crosslinked cells were then pelleted by centrifugation at 700 × *g* for 5 min at 4 °C. A total of 10^7^ cells were lysed in 1 mL SDS lysis buffer (100 mM NaCl, 50 mM Tris-HCl pH 8.1, 5 mM EDTA, 1% SDS, 3% Triton X and 1X Protease Inhibitor Cocktail II.). Cells were sheared with 4–5 sets of 10-second pulses on wet ice using a Sonicator with a 2 mm tip and set to 30% of maximum power gave the appropriate length DNA fragments. Cell lysate was centrifuged at a minimum of 10,000 × *g* but not exceeding 15,000 × *g* at 4 °C for 10 min to remove insoluble material. 100 μl supernatant was diluted with ChIP dilution buffer to 1000 μl before the addition of the antibody bead complexes. anti-FOXP1 (#4402T, Cell Signaling Technologies, 1:500), anti-TET2 (#36449, Cell Signaling Technology, 1:500), and Normal Mouse IgG antibodies (#12-371B, Millipore) was incubated with 60 μl of ChIP Blocked Protein G Agarose (# 16-201D, Millipore) for 1 h at 4 °C under rotation and then washed several times with ChIP dilution buffer to remove unbound antibodies before the addition of chromatin. After addition of chromatin, the mixtures were then incubated for 24 h under rotation at 4 °C. The beads containing the precipitated chromatin were then washed extensively with Low Salt Immune Complex Wash Buffer (#20-154, Millipore) once, High Salt Immune Complex Wash Buffer (#20-155, Millipore) once, LiCl Immune Complex Wash Buffer (#20-156, Millipore) once and TE buffer twice (#20-157, Millipore). The beads were then transferred to a new Eppendorf tubes and chromatin complex was eluted from the beads. A total 20% of the eluted chromatin was then retained as the primary ChIP. The remaining 80% of the eluted material was then used for the sequential ChIPs and was added to antibody bead complexes. The FOXP1 eluted chromatin was sequential ChIPed for TET2 by anti-TET2 (#36449, Cell Signaling Technology, 1:500) for 24 h under rotation at 4 °C. The beads containing the precipitated bivalent chromatin were then washed extensively in ChIP wash buffers 1–4, transferred to new Eppendorf tubes and de-crosslinked in 100 μl TE buffer pH 9.5 at 65 °C for 5 h, followed by 30 min of RNAse A treatment at 37 °C and 2 h Proteinase K treatment at 45 °C. The DNA was then purified from the solution via phenol chloroform precipitation overnight, lyophilized by a speed vacuum and resuspended in 11 μl of nuclease free dH_2_O. For real-time qPCR, total RNA was extracted from cells by using Direct-zol™ RNA MiniPrep Plus (Zymo Research). RNA was reverse-transcribed by using Superscript II kit (Invitrogen). The results were analyzed by the LightCycler96 (Roche, v1.1) and CFX96 ThermoCycler (Biorad) using PrimePCR SYBR Green Assay (Biorad), and quantification of cDNA levels was normalized to Actin as Ct (difference of cycling threshold) = Ct (target) – Ct (control). Customized primer sequences used in this study are listed in Supplementary Table 1.

### Detection of total 5hmc level by dot blot assay

Genomic DNA was denatured in 0.4 M NaOH, 10 mM EDTA at 99 °C for 5 min, and then neutralized by adding an equal volume of cold 2 M ammonium acetate (pH 7.0). Next, the denatured DNA sample (along with the 2-fold diluted sample) were spotted on an Amersham Hybond N+ membrane (GE Healthcare) and air dry. The membrane was then UV-linked and blocked with Blocking Solution 5% milk, PBST (1×PBS + 0.1%Tween-20) overnight at 4 °C. After blocking, the membrane was incubated with monoclonal 5-hmC antibody (#39769, Active Motif; 1:1000) and HRP-conjugated secondary antibody (#610-103-121, Rockland Immunochemicals, 1:5000) then visualized by SuperSignal West Dura Chemiluminescent Substrate (Thermo Scientific).

### Quantitation of 5hmC by methylation-sensitive qPCR

Genomic DNA was treated with T4 Phage β-glucosyltransferase (T4-BGT, New England Biolabs) and UDP-Glucose (UDP-Glc) according to the manufacturer’s instruction (EpiMark 5-hmC and 5-mC Analysis Kit, New England Biolabs). Glucosylated genomic DNA (100 ng) was digested with 10 U of HpaII, MspI or no enzyme (control group) at 37 °C overnight, followed by inactivation for 20 min at 80 °C. The HpaII- or MspI-resistant fraction was quantified by qPCR using primers designed around at least one HpaII/MspI site, and normalizing to the mock digestion control. The calculation of quantitation of 5-hydroxymethylcytosine at a specific CCGG Site follows the manufacturer’s instruction (EpiMark 5-hmC and 5-mC Analysis Kit, New England Biolabs).

### Sphere formation and 3D matrigel culture and acini staining

For serial sphere formation, 10^4^ cells were seeded in a 6-well low attachment cell culture plate and cultured in MammoCult medium (Stem Cell Technologies) for 5–7 days and serially passaged^10^. Frequency of sphere-forming cells were calculated by Extreme Limiting Dilution Analysis (WEHI Bioinformatic Resources Webtool 2014, http://bioinf.wehi.edu.au/software/elda/). For 3D differentiation culture, single cell suspension was subjected to 3D on-top matrigel culture (BD Biosciences) on 24-well plates at 20,000 cells/well density. Cells were incubated for 12 days, and the medium was replenished every 2 days. At the end of incubation, cells were fixed and subjected to immunofluorescence analysis. Cells were fixed using formalin for 20 min at room temperature (RT). Next, cells were permeabilized with 0.5% Triton X-100 in PBS for 5 min at RT and washed 3 times with 100 mM glycine at RT. Fixed cells were blocked for 1.5 h with 10% goat serum and then incubated with primary antibodies overnight at 4 °C. The primary antibodies used were used as follows: rat-anti-integrin-alpha6 (#MA5-16884, ThermoFisher Scientific, 1:200 in Dako antibody diluent buffer) and rabbit-anti-E-cadherin (#sc-8426, Santa Cruz, 1:100 in Dako antibody diluent buffer). Cells were incubated with secondary antibody for 1 h, followed by three washes at RT. Secondary antibodies were used as follows: Rhodamine Red-conjugated goat anti rabbit IgG (#111-295-003, Jackson Immunoresearch, 1:400) and FITC-conjugated goat anti mouse IgG (#115-095-003, Jackson Immunoresearch, 1:400). Cell nuclei were counterstained and mounted with Prolong Gold Antifade Reagent with DAPI (Molecular Probes) overnight at RT.

### Statistics and reproducibility

Each independent experiment was successfully performed with similar results at least three times. For phenotype analysis, the female animals were littermates housed in the same cage with synchronized estrous cycle. For drug treatment experiments, the female animal cohorts of the specific genotype and age were randomly allocated to tamoxifen or control vehicle treatment groups. Sample size was chosen based on power analysis (desired power = 80% and significance *p* < 0.05). Differences between individual groups were analyzed by two-tailed Student’s *t* test or by one-way ANOVA test for multiple group analysis. The dose–response curves were evaluated using GraphPad Prism to determine whether the curves were statistically different with respect to the fitted midpoints (log EC50) using the sum-of-squares *F* test. If not otherwise noted, no methods were used to determine whether the data met assumptions of the statistical approach; no inclusion/exclusion criteria/cases were applied. All analyses were carried out using Microsoft Excel 16.0 or GraphPad Prism 8.0 and presented as mean ± the standard deviation of the mean (SD). *P* value of 0.05 or lower were considered statistically significant for all experiments. The statistical parameters can be found in the figure legends.

## Supplementary information

Supplementary information

Description of Additional Supplementary Files

Supplementary dataset 1

Supplementary dataset 2

Supplementary dataset 3

## Data Availability

Genome wide bisulfite sequencing data were submitted to NCBI GEO repository (GSE147367). Data supporting the findings of this study are available within the article and its Supplementary information files and from the corresponding author upon reasonable request. Supplementary information provides Supplementary Figs. 1–4 and Supplementary Data 1–4. Source data file provides data underlying Figs. 1a, f–j, 2a–d, f, h–m, 3a–i, 4b, d, e, g, h, and Supplementary Figs. 1b, e–j, l–n, 2a, b, e, g–i, 3a–e. Source data are provided with this paper.

## Source data

Source data
