# Supplementary material for: TET2 directs mammary luminal cell differentiation and endocrine response
Source: Nat Commun. 2020 Sep 15;11:4642. doi: 10.1038/s41467-020-18129-w (PMC7493981; doi:10.1038/s41467-020-18129-w)
Supplement: Supplementary file 1 — Supplementary information [file 41467_2020_18129_MOESM1_ESM.pdf]

Supplementary Information

TET2 directs mammary luminal cell differentiation and endocrine response

Kim and Wu et al.

Supplementary Figure 1
(Related to Figure 1)

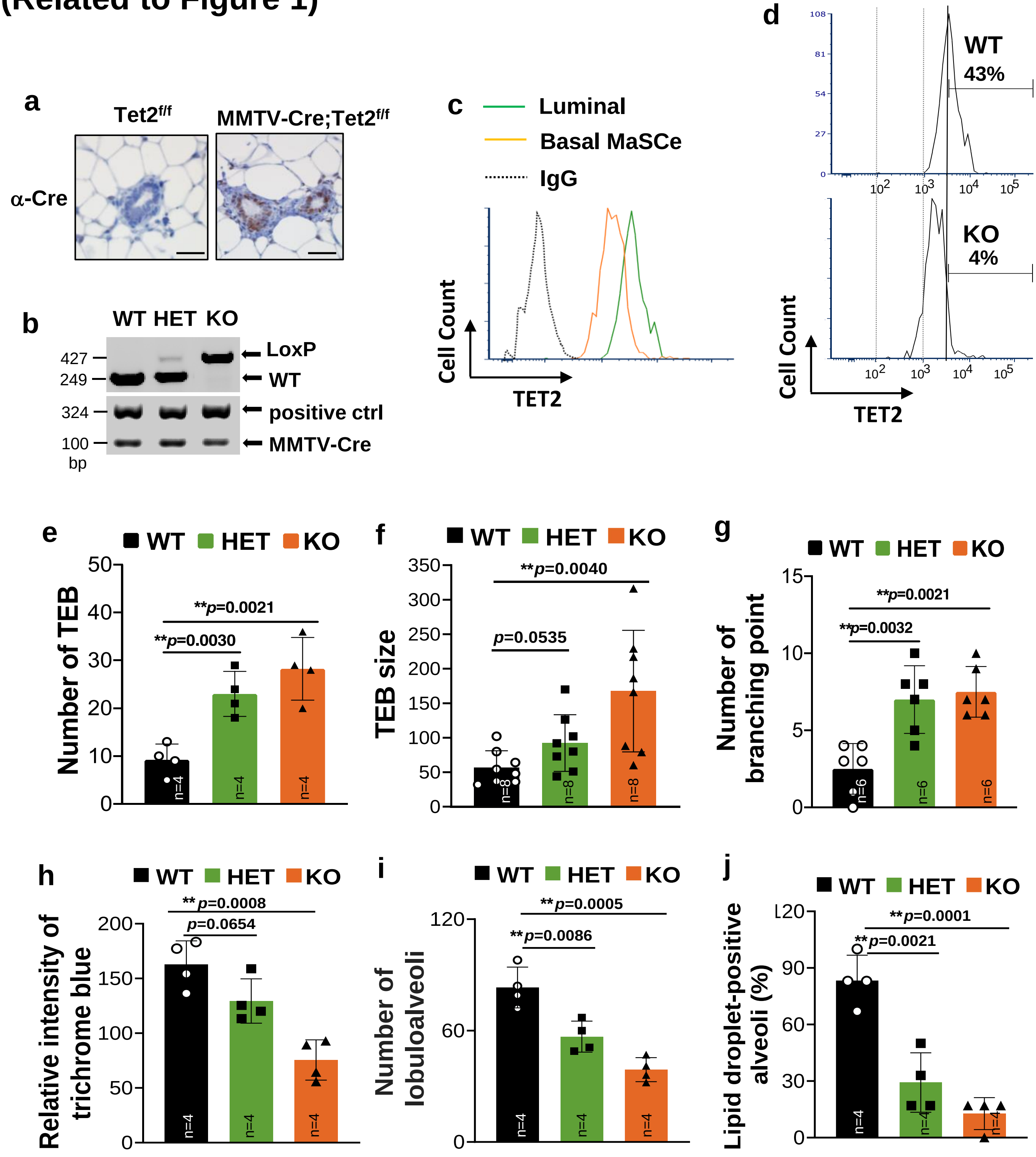

k

Cell #	WT	KO
**500**	5/5	5/5
**200**	1/5	4/5
**100**	0/5	3/5
**Frequency**	1/398	1/112

*Chi-Square *p=0.02*

Supplementary Figure 1 Cont.

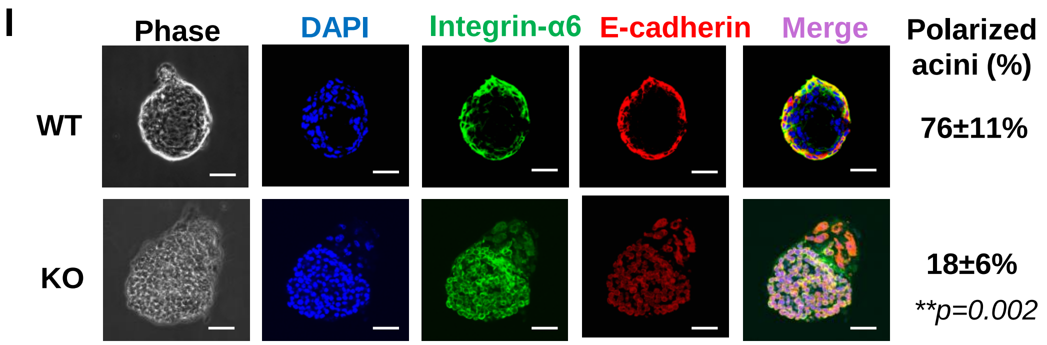

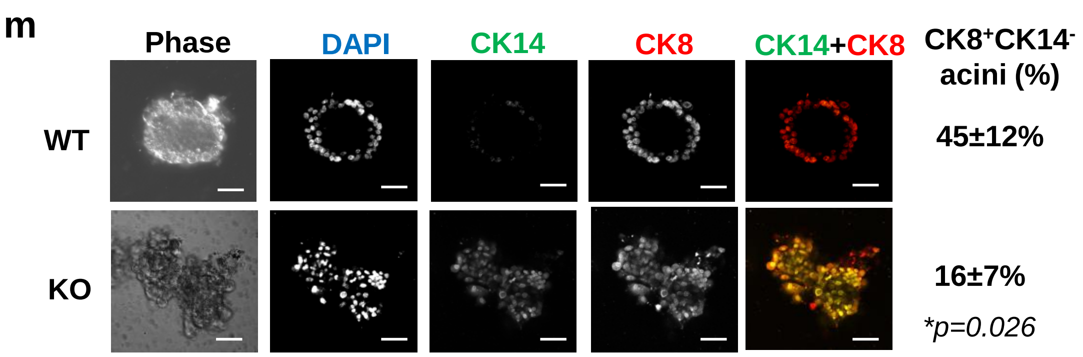

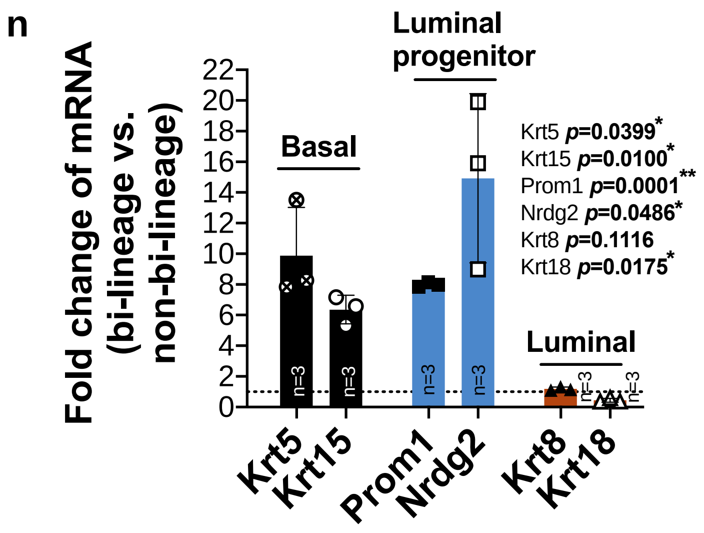

Supplementary Figure 1. Loss of TET2 enhances mammary stem cell enriched population and impairs luminal cell differentiation. (a) Immunostaining of Cre in mammary tissues from 7-week-old $Tet2^{f/f}$ and MMTV-Cre;$Tet2^{f/f}$ female mice (scale bar: 100 μm). **(b)** Verification of MMTV-Cre mediated Tet2 gene deletion using PCR genotyping. **(c)** Flow cytometry histogram showing TET2 expression in basal MaSC-enriched cell population (MaSCe) and luminal cell population (Lum) from 7-week-old WT female mouse mammary epithelial cells using intracellular staining. **(d)** Flow cytometry histogram showing TET2 expression in the luminal cell populations isolated from 7-week-old WT and KO female mouse mammary epithelial cells using intracellular staining; bar indicating percentage of cells with positive TET2 expression. **(e-h)** Bar graphs showing **(e)** number of TEB, **(f)** size of TEB, **(g)** number of ductal branching point, and **(h)** relative intensity of trichrome blue staining in collagen enriched tissue area from Figures 1b-1c. **(i-j)** Bar graphs showing **(i)** number of lobuloalveoli, and **(j)** percentage of lipid droplet-positive alveoli from Figure 1d. n=4 data points analyzed from four independent tissue section staining images of two animals for each group in **(e), (h), (i), (j)**. n=8 data points analyzed from eight independent tissue section staining images of two animals for each group in **(f)**. n=6 data points analyzed from six independent tissue section staining images of two animals for each group in **(g)**. Quantification of TEB size was determined by measuring the relative TEB area using ImageJ. Quantification of fibrosis was determined by measuring the relative tricrhome blue staining intensity over an equal vision field area using ImageJ Fiji. **(k)** In vitro limiting dilution analysis showing calculated frequency of sphere forming WT and KO MaSCs (n=5 independent experiments). Representative confocal immunofluorescence images of **(l)** acini stained for basal polarity marker (integrin-α6, green), epithelial cell marker (E-cadherin, red) and DAPI (nucleus, blue; scale bar: 50μm). The percentage of normal polarized acini was analyzed from 10-20 acini each field for each group (n=3 independent experiments). **(m)** Acini stained for basal cell lineage marker (CK14 green), luminal cell lineage marker (CK8, red) and DAPI (scale bar: 50μm). The percentage of $CK8^{+}CK14^{-}$ acini was analyzed from 10-20 acini each field for each group (n=3 independent experiments). **(n)** Fold change of gene expression of basal cell markers, luminal progenitor cell markers, and mature luminal cell markers compared between FACS-sorted bi-lineage mammary epithelial cell population ($CK14^{+}CK8^{+}$) with the non-bi-lineage population from mouse mammary epithelial cells (n=3 independent experiments). Data were presented as mean ±SD. p-values were determined by two-sided Student’s t-test **(e-j, l-m)** or two-sided one sample t-test **(n)**; asterisk indicates $p<0.05$, double asterisks indicate $p<0.01$. Source data are provided as a source data file.

Supplementary Figure 2 (Related to Figure 2)

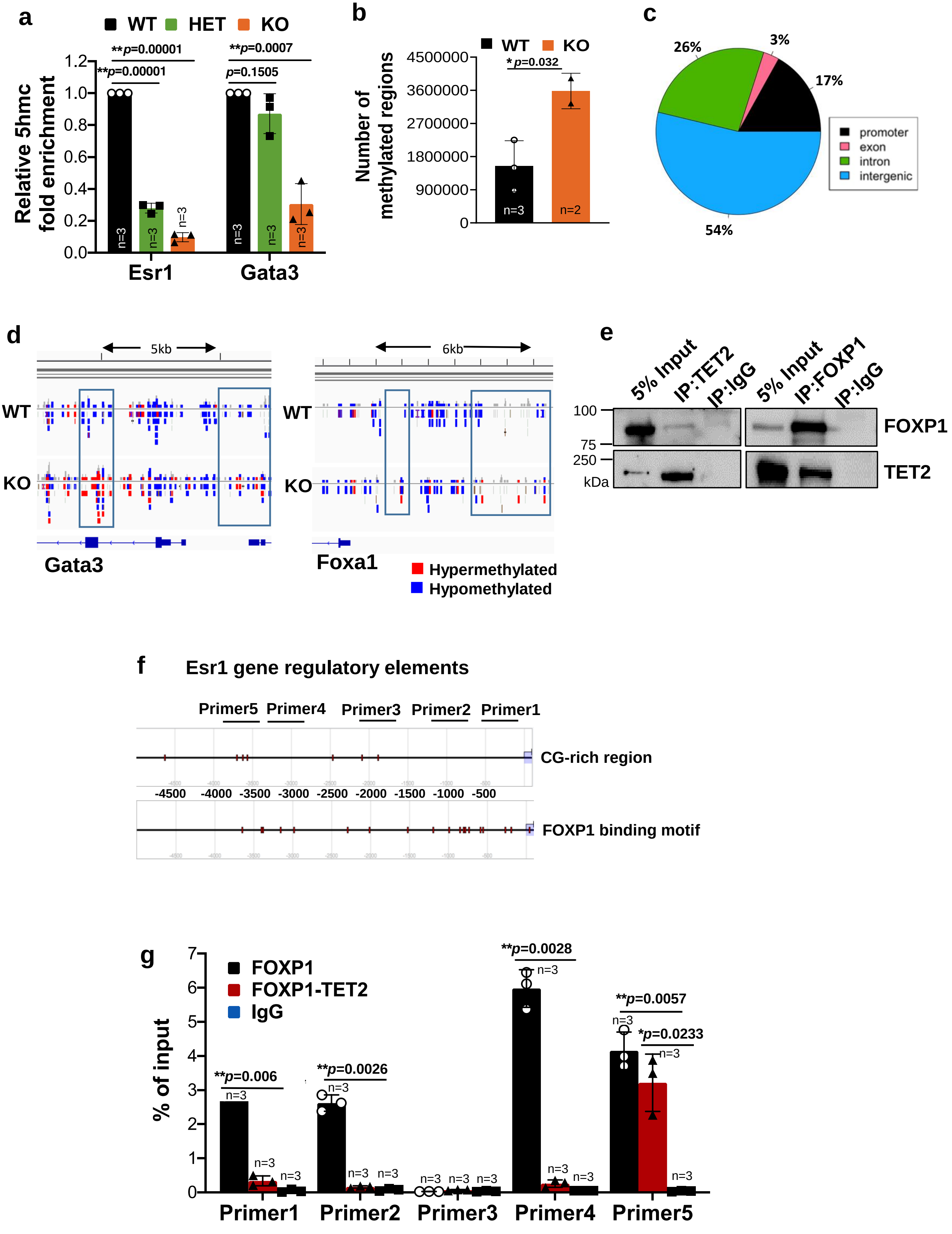

Supplementary Figure 2 Cont.

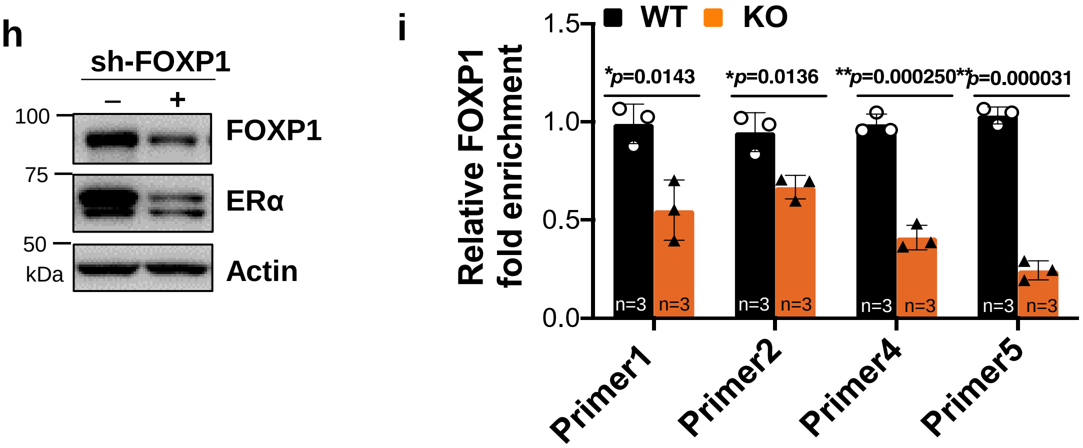

Supplementary Figure 2. TET2-FOXP1 complex mediates *Esr1* demethylation and ERα expression. (a) Fold enrichment of 5hmc in *Esr1* and *Gata3* genes from mammary epithelial cells of 7-week-old WT, HET, and KO female mice (n=3 animals/group). **(b)** The number of total methylated regions, **(c)** the percentage of differential methylated regions in the annotated genomic locations, and **(d)** methylation track providing visualization of DNA methylation status surrounding Gata3 and Foxa1 genes (box indicates differential methylation region), of biologically independent mammary epithelial cell samples from 7-week-old WT (n=3) and KO (n=2) female mice as shown in Figures 2e-2g. **(e)** Reciprocal co-immunoprecipitation showing endogenous FOXP1 and TET2 interaction in MCF12A cells. **(f)** Diagram showing putative GC-rich region and FOXP1 binding motif within the regulatory elements of *Esr1* gene (promoter and enhancer regions). **(g)** Bar graph showing fold enrichment of FOXP1 (by ChIP) or FOXP1-TET2 (by Sequential ChIP) at *Esr1* regulatory elements in WT and KO mammary epithelial cells (n=3 independent experiments). **(h)** ERα and FOXP1 Protein expression in MCF7 cells stably expressing sh-FOXP1 or the control vector. **(i)** Bar graph showing fold enrichment of FOXP1 at the indicated *Esr1 r*egulatory elements in WT and KO mammary epithelial cells (n=3 independent experiments). Data were presented as mean ±SD. p-values were determined by two-sided Student's t-test; asterisk indicates $p<0.05$, double asterisks indicate $p<0.01$. Source data are provided as a source data file.

Supplementary Figure 3 (Related to Figure 4)

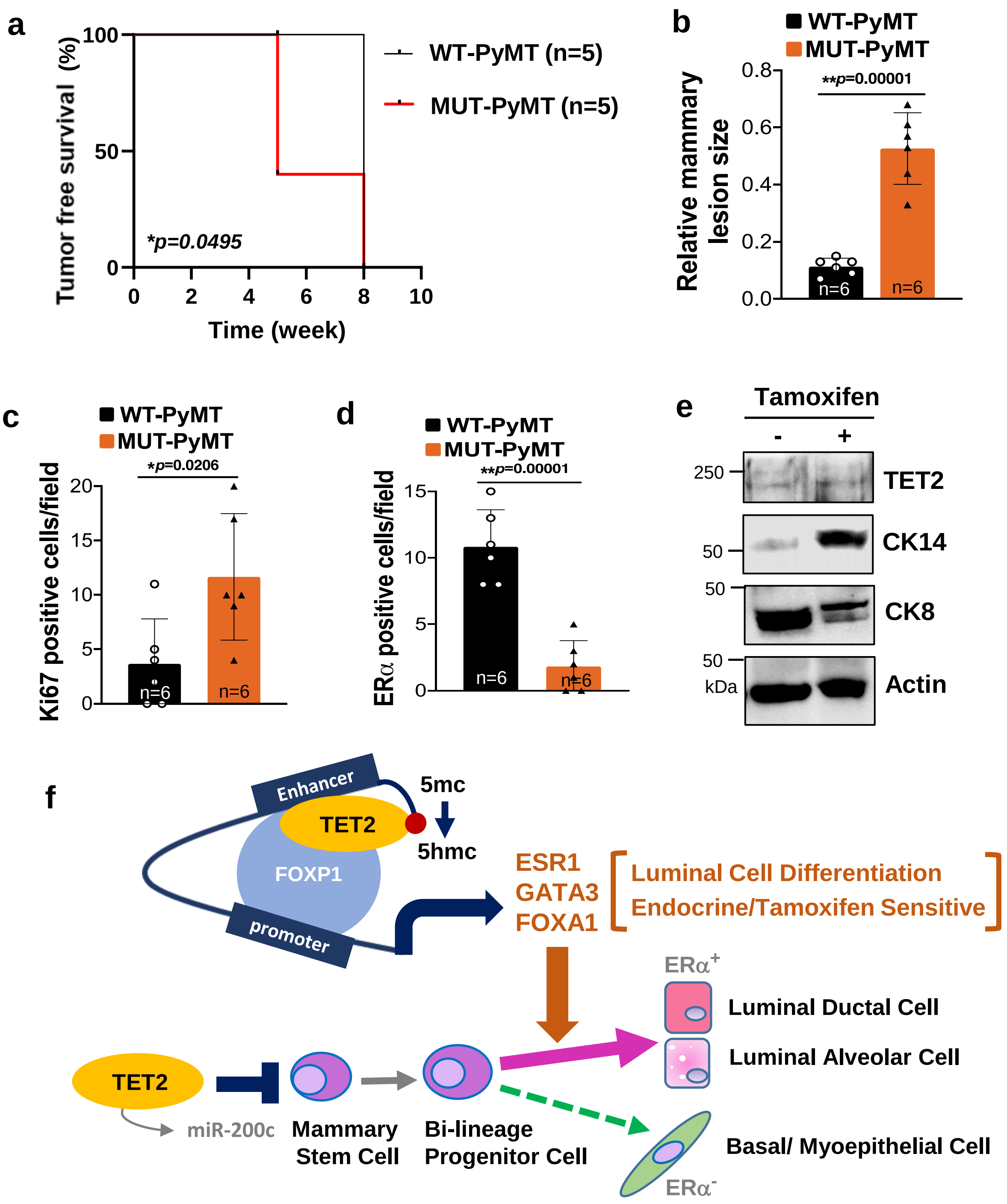

Supplementary Figure 3. TET2 plays a role in maintaining mammary epithelial homeostasis and endocrine sensitivity. (a) Tumor free survival of WT-PyMT and MUT-PyMT animals (n= 5 animals/group). p-value was determined by two-sided Log-rank test. **(b-d)** Bar graphs showing **(b)** relative mammary lesion size, **(c)** number of Ki67 positive cells, and **(d)** number of ERα positive cells per vision field from Figure 4a. n=6 data points analyzed from six independent tissue section staining images of two animals for each group. Quantification of mammary lesion size was determined by measuring the relative lesion area over an equal vision field area using ImageJ. Data were presented as mean ±SD. p-values were determined by two-sided Student's t-test; asterisk indicates $p<0.05$, double asterisks indicate $p<0.01$. **(e)** Protein expression of the residual mammary tumor tissue from a tamoxifen-treated WT mouse. **(f)** A proposed model of the role of TET2 in regulation of luminal cell differentiation and endocrine response. Source data are provided as a source data file.

Supplementary Figure 4

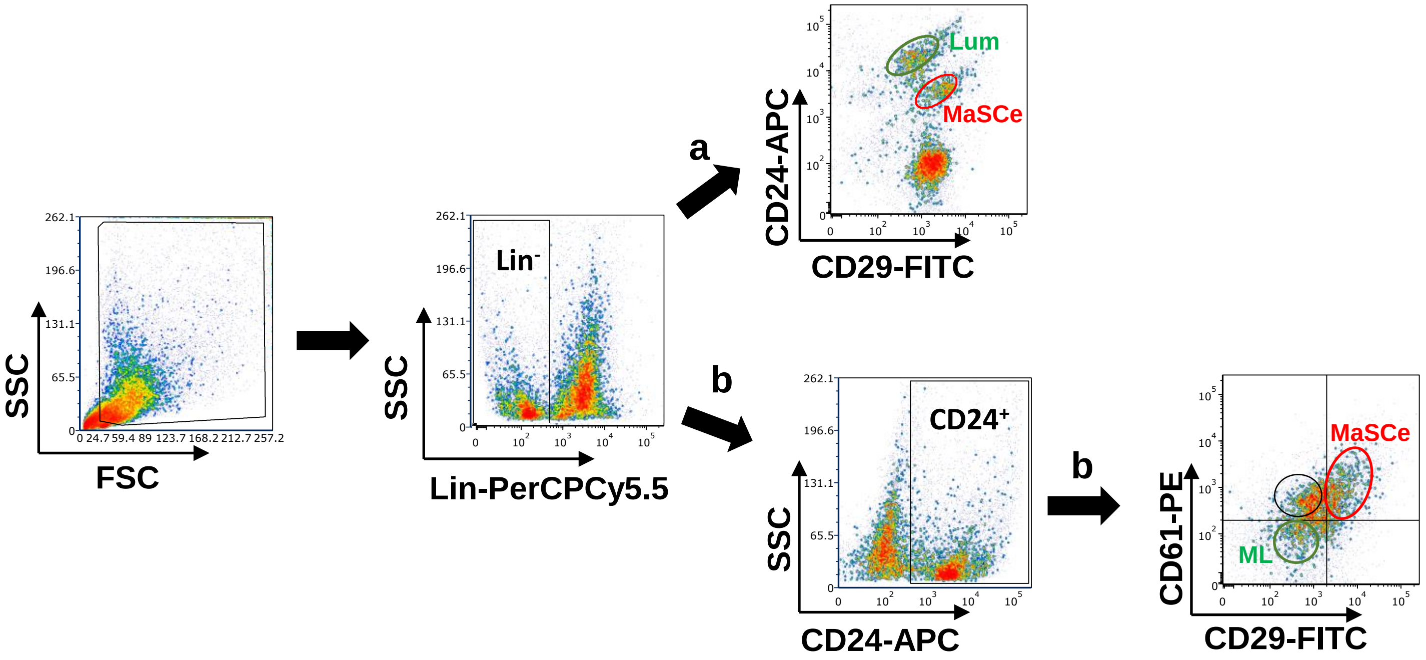

Supplementary Figure 4. Gating strategies used for FACS analysis of mammary epithelial cell sub-populations presented in Figures 1g, 1h, 4b, and Supplementary Figure 1c. Gating panel (a) corresponds to Figures 1g and 4b, Supplementary Figure 1c. Gating panel (b) corresponds to Figure 1h.

Supplementary Table 1

Primer name	Sequence	Experiment
mFoxa1-ChIP-F	CCTCCAAGGCACCGCC	ChIP-QPCR
mFoxa1-ChIP-R	ATCCCCTCCCCCAAACCTG	ChIP-QPCR
mGata3-ChIP-F	TCCAAGTTTTGAATAAGTGGGAAAT	ChIP-QPCR
mGata3-ChIP-R	AGCCTGGGTCCAGAAAACAC	ChIP-QPCR
mEsr1-ChIP-F	ACAAAACACCAGATTAAAGCCCC	ChIP-QPCR
mEsr1-ChIP-R	TGGCTCAGCAGTTCTTGTCA	ChIP-QPCR
hGATA3-F	GCCCCTCATTAAGCCCAAG	QRT-PCR
hGATA3-R	TTGTGGTGGTCTGACAGTTCG	QRT-PCR
mGata3-F	CTCGGCCATTCGTACATGGAA	QRT-PCR
mGata3-R	GGATACCTCTGCACCGTAGC	QRT-PCR
hFOXA1-F	GCAATACTCGCCTTACGGCT	QRT-PCR
hFOXA1-R	TACACACCTTGGTAGTACGCC	QRT-PCR
mFoxa1-F	ATGAGAGCAACGACTGGAACA	QRT-PCR
mFoxa1-R	TCATGGAGTTCATAGAGCCCA	QRT-PCR
hESR1-F	AGAGGGAAAGTAGGGCAGAA	QRT-PCR
hESR1-R	TGGGAAATGAAGAAGAGCTG	QRT-PCR
mEsr1-F	TTGTGTGCCTCAAATCCATC	QRT-PCR
mEsr1-R	GAGATGCTCCATGCCTTTGT	QRT-PCR
hACTB-F	GTGGGCATGGGTCAGAAG	QRT-PCR
hACTB-R	TCCATCACGATGCCAGTG	QRT-PCR
mActb-F	AGTGTGACGTTGACATCCGT	QRT-PCR
mActb-R	TGCTAGGAGCCAGAGCAGTA	QRT-PCR
hGREB1-F	GGCAGGACCAGCTTCTGA	QRT-PCR
hGREB1-R	CTGTTCCCACCACCTTGG	QRT-PCR
mGreb1-F	CTGCATCGTCATCCCTCTCC	QRT-PCR
mGreb1-R	TAGACCCCTGACTCACTCGG	QRT-PCR
hPGR-F	GTGCCTATCCTGCCTCTCAATC	QRT-PCR
hPGR-R	CCCGCCGTCGTAACTTTCG	QRT-PCR
mPgr-F	TGGACTCAGGTCCCTTCCAA	QRT-PCR
mPgr-R	CCTTGATCTCTGGCCGACTC	QRT-PCR

Supplementary Table 1. List of primer sequences used in this study.

nature research

Corresponding author(s): Alice Chang

Last updated by author(s): 07/22/2020

Reporting Summary

Nature Research wishes to improve the reproducibility of the work that we publish. This form provides structure for consistency and transparency in reporting. For further information on Nature Research policies, see Authors & Referees and the Editorial Policy Checklist.

Statistics

For all statistical analyses, confirm that the following items are present in the figure legend, table legend, main text, or Methods section.

n/a	Confirmed	
☐	☒	The exact sample size (n) for each experimental group/condition, given as a discrete number and unit of measurement
☒	☐	A statement on whether measurements were taken from distinct samples or whether the same sample was measured repeatedly
☐	☒	The statistical test(s) used AND whether they are one- or two-sided<br>*Only common tests should be described solely by name; describe more complex techniques in the Methods section.*
☒	☐	A description of all covariates tested
☐	☒	A description of any assumptions or corrections, such as tests of normality and adjustment for multiple comparisons
☐	☒	A full description of the statistical parameters including central tendency (e.g. means) or other basic estimates (e.g. regression coefficient) AND variation (e.g. standard deviation) or associated estimates of uncertainty (e.g. confidence intervals)
☐	☒	For null hypothesis testing, the test statistic (e.g. F, t, r) with confidence intervals, effect sizes, degrees of freedom and P value noted<br>*Give P values as exact values whenever suitable.*
☒	☐	For Bayesian analysis, information on the choice of priors and Markov chain Monte Carlo settings
☒	☐	For hierarchical and complex designs, identification of the appropriate level for tests and full reporting of outcomes
☒	☐	Estimates of effect sizes (e.g. Cohen's d, Pearson's r), indicating how they were calculated

Our web collection on statistics for biologists contains articles on many of the points above.

Software and code

Policy information about availability of computer code

Data collection	BD FACS Diva 8, Olympus Fluoview Fv10i (v3.0), LightCycler96 (v1.1)
Data analysis	GraphPad Prism 8, Microsoft Office365 Excel (v16.0), ImageJ (v1.4.3.67), ELDA software (WEHI Bioinformatic Resources Webtool 2014), Integrative Genomics Viewer (v2.8.0), Bismark (v0.203.0), FCS express 6 (Denovo Software), FASTQC (v0.11.8), Trim Galore (v0.5.0), Homer software (sv4.11.1), methylation calling and alignment (Bismark Bowtie2)

For manuscripts utilizing custom algorithms or software that are central to the research but not yet described in published literature, software must be made available to editors/reviewers. We strongly encourage code deposition in a community repository (e.g. GitHub). See the Nature Research guidelines for submitting code & software for further information.

Data

Policy information about availability of data

All manuscripts must include a data availability statement. This statement should provide the following information, where applicable:

- Accession codes, unique identifiers, or web links for publicly available datasets
- A list of figures that have associated raw data
- A description of any restrictions on data availability

Genome wide bisulfite sequencing data was submitted to NCBI GEO repository (GSE147367). Data supporting the findings of this study are available within the article and its supplementary information files and from the corresponding author upon reasonable request. Supplementary Information provides Supplementary Figures 1-4 and Supplementary Data 1-4. Source Data file provides data underlying Figures 1A, 1F-1J, 2A-2D, 2F, 2H-2M, 3A-3I, 4B, 4D, 4E, 4G, 4H, and Supplementary Figures 1B, 1E-1J, 1L-1N, 2A, 2B, 2E, 2G-2I, 3A-3E.

Field-specific reporting

Please select the one below that is the best fit for your research. If you are not sure, read the appropriate sections before making your selection.

☒ Life sciences ☐ Behavioural & social sciences ☐ Ecological, evolutionary & environmental sciences

For a reference copy of the document with all sections, see nature.com/documents/nr-reporting-summary-flat.pdf

Life sciences study design

All studies must disclose on these points even when the disclosure is negative.

Sample size	The sample sizes in each experimental group was determined from our preliminary experiments and based on 80% power and two- sided tests for 5% level of significance.
Data exclusions	All samples that met adequate experimental conditions were included in the analysis; no methods were used to determine whether the data met assumptions of the statistical approach; no inclusion/exclusion criteria/cases were applied.
Replication	Experiments were successfully performed for at least 3 times and/or with sufficient animals per group to demonstrate statistical significance as specified in the figure legend.
Randomization	Only female animals were used for the purpose of studying mammary gland development and breast cancer implication. The female animals were age matched litter mates housed in the same cage with synchronized estrous cycle. Cell dishes for different treatments was allocated by randomization.
Blinding	Investigators were not blinded during data collection because sample label contained treatment. Sample analysis was performed based on consistent criteria. Blinding was not performed in animal expriments as animals were genotyped and labeled.

Reporting for specific materials, systems and methods

We require information from authors about some types of materials, experimental systems and methods used in many studies. Here, indicate whether each material, system or method listed is relevant to your study. If you are not sure if a list item applies to your research, read the appropriate section before selecting a response.

Materials & experimental systems

n/a	Involved in the study	
☐	☒	Antibodies
☐	☒	Eukaryotic cell lines
☒	☐	Palaeontology
☐	☒	Animals and other organisms
☒	☐	Human research participants
☒	☐	Clinical data

Methods

n/a	Involved in the study	
☒	☐	ChIP-seq
☐	☒	Flow cytometry
☒	☐	MRI-based neuroimaging

Antibodies

Antibodies used

Immunohistochemistry staining: anti-TET2 (#ABE364, Millipore Sigma, 1:250), anti-Cre (#15036, Cell Signaling, 1:200), anit-Ki67 (#PIPA519462, Invitrogen, 1:200), and anti-ERa (#ab32063, Abcam, 1:100).

Immunofluorescence staining: anti-CK8 (#ab59400, Abcam, 1:250), anti-CK14 (#ab7800, Abcam, 1:250), anti-MUC1 (#ab45167, Abcam, 1:250), anti-a-SMA (#A5228, Sigma Aldrich, 1:500), rabbit-anti-E-cadherin (#sc-8426, Santa Cruz, 1:100), rat-anti-integrin-alpha6 ((#MA5-16884, ThermoFisher Scientific, 1:200), Rhodamine Red-conjugated goat anti rabbit IgG (#111-295-003, Jackson Immunoresearch, 1:400), FITC-conjugated goat anti mouse IgG (#115-095-003, Jackson Immunoresearch, 1:400)

Flow cytometry: FITC-conjugated anti-CD29 (#561796, BD Biosciences, 1:100), PE-Cy7-conjugated CD29, eBioscience, 1:100), PE-conjugated anti-CD29 (#25029180, eBioscience, 1:100), APC-conjugated anti-CD24 (#562349, BD Biosciences, 1:100), PE-conjugate anti-CD24 (#553262, BD Biosciences, 1:100), PE-conjugated anti-CD61 (#561910, BD Biosciences, 1:100), PerCP-Cy™5.5 Mouse Lineage Antibody Cocktail (#561317, BD Biosciences, 1:100), anti-TET2 (#36449, Cell Signaling Technology, 1:100), Alexa Fluor® 594-conjugated anti-Cytokeratin 8 (#NB120-9287AF594, Novus Biologicals, 1:200), Alexa Fluor® 647- conjugated anti-Cytokeratin 14 (#NBP2-47720AF647, Novus Biologicals, 1:200) and Alexa Fluor® 488-conjugated Anti-rabbit IgG (H+L) F(ab')2 Fragment (#A-11070, ThermoFisher Scientific, 1:500)

Immunoblotting: anti-TET2 (#36449, Cell Signaling Technology; #61389, Active Motif, 1:1000), anti-b-Actin (#A5316, Sigma, 1:5000), anti-b-Casein (#sc166530, Santa Cruz, 1:1000), anti-ERa (#ab32063, Abcam, 1:1000), anti-GATA3 (#PA520892, Thermo Fisher Scientific, 1:1000), anti-FOXA1 (sc101058, Santa Cruz, 1:1000), and anti-FOXP1 (#4402T, Cell Signaling Technologies, 1:1000), 5-hmC antibody ((#39769, Active Motif; 1:1000), HRP-conjugated secondary antibody (#610-103-121 and 610-103-122,

Rockland Immunochemicals, 1:5000)

Chromatin immunoprecipitation: anti-FOXP1 (#4402T, Cell Signaling Technologies, 1:500), anti-TET2 (#92529, Cell Signaling Technology, 1:500), and Normal Rabbit IgG antibodies (#12-370, Millipore, 1:500)

Validation

Antibodies have validation statements/data on the manufacturer's website/data sheet. Please refer to the web pages below:
Immunohistochemistry staining:
1. anti-TET2 (#ABE364, Millipore Sigma, 1:250)
Species: Human, Rat, Mouse; Application: WB, IHC(P), ICC
Website: https://www.emdmillipore.com/US/en/product/Anti-Tet2-Antibody,MM_NF-ABE364
2. anti-Cre (#15036, Cell Signaling, 1:200)
Species: CRE; Application: WB, IHC, ICC
Website: https://www.cellsignal.com/products/primary-antibodies/cre-recombinase-d7l7l-xp-rabbit-mab/15036
3. anit-Ki67 (#PIPA519462, Invitrogen, 1:200)
Species: Bovine, Dog, Deer, Horse, Hamster, Human, Mouse, Non-human primate, Sheep, Pig, Rabbit, Rat; Application: IHC(P), ICC/IF
Website: https://www.thermofisher.com/antibody/product/Ki-67-Antibody-Polyclonal/PA5-19462
4. anti-ERa (#ab32063, Abcam, 1:100)
Species: Human, Rat, Mouse; Application: ICC/IF, Flow Cyt, ChIP, WB, IHC(P)
Website: https://www.abcam.com/estrogen-receptor-alpha-antibody-e115-chip-grade-ab32063.html

Immunofluorescence staining:
1. anti-CK8 (#ab59400, Abcam, 1:250)
Species: Human, Rat, Mouse; Application: ICC/IF, IHC-Fr, WB, IHC-P, ELISA
Website: https://www.abcam.com/cytokeratin-8-antibody-ab59400.html
2. anti-CK14 (#ab7800, Abcam, 1:250)
Species: Human, Rat, Mouse; Application: WB, Flow Cyt, ICC/IF, IHC-P
Website: https://www.abcam.com/cytokeratin-14-antibody-ll002-ab7800.html
3. anti-MUC1 (#ab45167, Abcam, 1:250)
Species: Human, Rat, Mouse; Application: IHC-Fr, WB, Flow Cyt, IP, ICC/IF
Website: https://www.abcam.com/muc1-antibody-ep1024y-ab45167.html
4. anti-a-SMA (#A5228, Sigma Aldrich, 1:500)
Species: human, frog, sheep, chicken, goat, bovine, rat, guinea pig, mouse, canine, rabbit, snake
; Application: IHC-Fr/P, WB, ICC/IF, ELISA
Website: https://www.sigmaaldrich.com/catalog/product/sigma/a5228?lang=en&region=US
5. rabbit-anti-E-cadherin (#sc-8426, Santa Cruz, 1:100)
Species: Human, Rat, Mouse; Application: WB, IP, IF, IHC(P) and ELISA
Website: https://www.scbt.com/p/e-cadherin-antibody-g-10
6. rat-anti-integrin-alpha6 (#MA5-16884, ThermoFisher Scientific, 1:200)
Species: Dog, Human, Mouse, Non-human primate, Sheep, Pig
; Application: IHC-Fr, Flow Cyt, IP, ICC/IF
Website: https://www.thermofisher.com/antibody/product/MA5-16884.html?CID=AFLBC-MA5-16884

Flow cytometry:
1. FITC-conjugated anti-CD29 (#561796, BD Biosciences, 1:100)
Species: Rat, Mouse; Application: Flow Cyt
Website: https://www.bdbiosciences.com/us/applications/research/stem-cell-research/cancer-research/mouse/fitc-hamster-anti-rat-cd29-ha25/p/561796
2. PE-Cy7-conjugated CD29, (#25029182, eBioscience, 1:100)
Species: Rat, Mouse; Application: Flow Cyt
Website: https://www.thermofisher.com/antibody/product/CD29-Integrin-beta-1-Antibody-clone-eBioHMb1-1-HMb1-1-Monoclonal/25-0291-82
3. PE-conjugated anti-CD29 (#25029180, eBioscience, 1:100)
Species: Rat, Mouse; Application: Flow Cyt
Website: https://www.thermofisher.com/antibody/product/CD29-Integrin-beta-1-Antibody-clone-eBioHMb1-1-HMb1-1-Monoclonal/12-0291-82
4. APC-conjugated anti-CD24 (#562349, BD Biosciences, 1:100)
Species: Mouse; Application: Flow Cyt
Website: https://www.bdbiosciences.com/us/applications/research/stem-cell-research/cancer-research/mouse/apc-rat-anti-mouse-cd24-m169/p/562349
5. PE-conjugate anti-CD24 (#553262, BD Biosciences, 1:100)
Species: Mouse; Application: Flow Cyt
Website: https://www.bdbiosciences.com/us/applications/research/stem-cell-research/cancer-research/mouse/pe-rat-anti-mouse-cd24-m169/p/553262
6. PE-conjugated anti-CD61 (#561910, BD Biosciences, 1:100)
Species: Mouse; Application: Flow Cyt
Website: https://www.bdbiosciences.com/us/reagents/research/antibodies-buffers/immunology-reagents/anti-mouse-antibodies/cell-surface-antigens/pe-hamster-anti-mouse-cd61-2c9g2-also-known-as-hm3-1/p/561910
7. PerCP-Cy™5.5 Mouse Lineage Antibody Cocktail (#561317, BD Biosciences, 1:100)
Species: Mouse; Application: Flow Cyt
Website: https://www.bdbiosciences.com/us/applications/research/stem-cell-research/stem-cell-kits-and-cocktails/mouse/percp-cy55-mouse-lineage-antibody-cocktail-with-isotype-control/p/561317
8. anti-TET2 (#36449, Cell Signaling Technology, 1:100)

Species: Mouse; Application: WB, IP, IF, Flow Cyt
Website: https://www.cellsignal.com/products/primary-antibodies/tet2-d6c7k-rabbit-mab-mouse-specific/36449
9. Alexa Fluor® 594-conjugated anti-Cytokeratin 8 (#NB120-9287AF594, Novus Biologicals, 1:200)
Species: Hu, Mu, Rt, Bv, Ch, Pm, Rb; Application: WB, Flow, IHC, IHC-Fr, IHC-P
Website: https://www.novusbio.com/products/cytokeratin-8-antibody-ae3_nb120-9287af594
10. Alexa Fluor® 647- conjugated anti-Cytokeratin 14 (#NBP2-47720AF647, Novus Biologicals, 1:200)
Species: Human, Rat, Mouse; Application: Flow, IHC, IHC-P
Website: https://www.novusbio.com/products/cytokeratin-14-antibody-krt14-532_nbp2-47720af647

Immunoblotting:
1. anti-TET2 (#36449, Cell Signaling Technology, 1:100)
Species: Mouse; Application: WB, IP, IF, Flow Cyt
Website: https://www.cellsignal.com/products/primary-antibodies/tet2-d6c7k-rabbit-mab-mouse-specific/36449
2. anti-TET2 (#61389, Active Motif, 1:1000)
Species: human; Application: WB, IP, IF, ICC
Website: https://www.activemotif.com/catalog/details/61389
3. anti-b-Actin (#A5316, Sigma, 1:5000)
Species: Drosophila, Hirudo medicinalis, carp, rabbit, wide range, pig, cat, human, rat, chicken, guinea pig, sheep, mouse, bovine, canine
; Application: WB, IF, IHC(P) and ELISA
Website: https://www.sigmaaldrich.com/catalog/product/sigma/a5316?lang=en&region=US
4. anti-b-Casein (#sc166530, Santa Cruz, 1:1000)
Species: Human, Rat, Mouse; Application: WB, IP, IF, IHC(P) and ELISA
Website: https://www.scbt.com/p/beta-casein-antibody-h-4
5. anti-ERa (#ab32063, Abcam, 1:1000)
Species: Human, Rat, Mouse; Application: ICC/IF, Flow Cyt, ChIP, WB, IHC-P
Website: https://www.abcam.com/estrogen-receptor-alpha-antibody-e115-chip-grade-ab32063.html
6. anti-GATA3 (#PA520892, Thermo Fisher Scientific, 1:1000)
Species: Human, Rat, Mouse; Application: WB, IHC, IF/ICC, ChIP
Website: https://www.thermofisher.com/antibody/product/GATA3-Antibody-Polyclonal/PA5-20892
7. anti-FOXA1 (sc101058, Santa Cruz, 1:1000)
Species: Human, Rat, Mouse; Application: WB, IP, IF, IHC(P), ELISA
Website: https://www.scbt.com/p/hnf-3alpha-antibody-q-6
8. anti-FOXP1 (#4402T, Cell Signaling Technologies, 1:1000)
Species: Human, Rat, Mouse; Application: WB, IP, IF, IHC and ChIP
Website: https://www.cellsignal.com/products/primary-antibodies/foxp1-d35d10-xp-rabbit-mab/4402
9. 5-hmC antibody (#39769, Active Motif; 1:1000)
Species: Human, Mouse, Not Species Specific; Application: MeDIP, IF/ICC, IHC, Flow Cyt, Dotblot
Website: https://www.activemotif.com/catalog/details/39769

Chromatin immunoprecipitation:
1. anti-FOXP1 (#4402T, Cell Signaling Technologies, 1:1000)
Species: Human, Rat, Mouse; Application: WB, IP, IF, IHC and ChIP
Website: https://www.cellsignal.com/products/primary-antibodies/foxp1-d35d10-xp-rabbit-mab/4402
2. anti-TET2 (#92529, Cell Signaling Technology, 1:500)
Species: Mouse; Application: WB, ChIP
Website: https://www.cellsignal.com/products/primary-antibodies/tet2-d9k3e-rabbit-mab-mouse-specific/92529

Eukaryotic cell lines

Policy information about cell lines

Cell line source(s)	MCF7, MCF12A, and 293T cell lines were purchased from American Type Culture Collection (ATCC).
Authentication	MCF7, MC F12A, and 293T cells were were authenticated by ATCC using Short Tandem Repeat (STR) profiling.
Mycoplasma contamination	MCF7, MCF12A, and 293T cells were tested as mycoplasma-free.
Commonly misidentified lines (See ICLAC register)	There are no commonly misidentified cell lines used in this study.

Animals and other organisms

Policy information about studies involving animals; ARRIVE guidelines recommended for reporting animal research

Laboratory animals	MMTV-Cre;Tet2+/+, MMTV-Cre;Tet2f/+, MMTV-Cre;Tet2f/f, Tet2f/+;PyMT, and MMTV-Cre;Tet2f/+;PyMT female mice were generated in this study; the age and the number of these animals were used as specified for experiments.
Wild animals	No wild animals were used in the study.

Field-collected samples	No Field-collected samples were used in the study.
Ethics oversight	Experiments were conducted with approval of the Animal Care and Use Committees at Purdue University and Roswell Park Comprehensive Cancer Center.

Note that full information on the approval of the study protocol must also be provided in the manuscript.

Flow Cytometry

Plots

Confirm that:

- [x] The axis labels state the marker and fluorochrome used (e.g. CD4-FITC).
- [x] The axis scales are clearly visible. Include numbers along axes only for bottom left plot of group (a 'group' is an analysis of identical markers).
- [x] All plots are contour plots with outliers or pseudocolor plots.
- [x] A numerical value for number of cells or percentage (with statistics) is provided.

Methodology

Sample preparation	Mammary epithelial cells were isolated from the mouse mammary gland and prepared and stained as described in Method.
Instrument	BD FACSAria Fusion, BD FACSCantoII
Software	FCS express 6 (Denovo Software)
Cell population abundance	10,000 cells to 100,000 cells were acquired per sample, the total population was analyzed.
Gating strategy	The gating boundaries were determined by using fsc/ssc and mouse linage marker cocktail as shown in Supplementary Fig S4.

- [x] Tick this box to confirm that a figure exemplifying the gating strategy is provided in the Supplementary Information.
