# Supplementary material for: TET2 directs mammary luminal cell differentiation and endocrine response
Source: Nat Commun. 2020 Sep 15;11:4642. doi: 10.1038/s41467-020-18129-w (PMC7493981; doi:10.1038/s41467-020-18129-w)
Supplement: Supplementary file 2 — Description of Additional Supplementary Files [file 41467_2020_18129_MOESM2_ESM.pdf]

Description of Additional Supplementary Files

Title: Supplementary Data 1

Description: Gene ontology (GO) analysis of the differentially methylated genes

Title: Supplementary Data 2

Description: List of the common transcription factor binding sites in the promoter regions of ESR1, GATA3, and FOXA1 genes

Title: Supplementary Data 3

Description: Motif analysis of the differentially methylated DNA regions
